# Abnormal distraction and load‐specific connectivity during working memory in cognitively normal Parkinson's disease

**DOI:** 10.1002/hbm.24868

**Published:** 2019-11-18

**Authors:** Deborah L. Harrington, Qian Shen, Julian Vincent Filoteo, Irene Litvan, Mingxiong Huang, Gabriel N. Castillo, Roland R. Lee, Ece Bayram

**Affiliations:** ^1^ Research, Radiology, and Psychology Services VA San Diego Healthcare System San Diego California; ^2^ Department of Radiology University of California San Diego California; ^3^ Department of Psychiatry University of California San Diego California; ^4^ Department of Neurosciences University of California San Diego California

**Keywords:** context‐dependent functional connectivity, distraction, fMRI, memory load, Parkinson's disease, visuospatial, working memory

## Abstract

Visuospatial working memory impairments are common in Parkinson's disease (PD), yet the underlying neural mechanisms are poorly understood. The present study investigated abnormalities in context‐dependent functional connectivity of working memory hubs in PD. Cognitively normal PD and control participants underwent fMRI while performing a visuospatial working memory task. To identify sources of dysfunction, distraction, and load‐modulated connectivity were disentangled for encoding and retrieval phases of the task. Despite normal working memory performance in PD, two features of abnormal connectivity were observed, one due to a loss in normal context‐related connectivity and another related to upregulated connectivity of hubs for which the controls did not exhibit context‐dependent connectivity. During encoding, striatal‐prefrontal coupling was lost in PD, both during distraction and high memory loads. However, long‐range connectivity of prefrontal, medial temporal and occipital hubs was upregulated in a context‐specific manner. Memory retrieval was characterized by different aberrant connectivity patterns, wherein precuneus connectivity was upregulated during distraction, whereas prefrontal couplings were lost as memory load approached capacity limits. Features of abnormal functional connectivity in PD had pathological and compensatory influences as they correlated with poorer working memory or better visuospatial skills. The results offer new insights into working memory‐related signatures of aberrant cortico–cortical and corticostriatal functional connections, which may portend future declines in different facets of working memory.

## 1 INTRODUCTION

Executive dysfunction is the most widely reported cognitive disturbance in Parkinson's disease (PD) (Muslimovic, Post, Speelman, & Schmand, 2005). A cornerstone of executive functioning is working memory (WM), which is impaired in PD more so for visuospatial than verbal information (Siegert, Weatherall, Taylor, & Abernethy, 2008). Visuospatial WM supports many everyday activities that require updating and processing of visuospatial information in continuously changing situations, and often in the face of distraction. For example, impaired driving and navigation in PD is associated with deficient updating (Ranchet, Paire‐Ficout, Marin‐Lamellet, Laurent, & Broussolle, 2011), visuospatial WM (Stolwyk, Charlton, Triggs, Iansek, & Bradshaw, 2006), and suppression of distractors (Uc et al., 2006). Despite numerous behavioral studies in PD (Siegert et al., 2008), neuroimaging studies have not characterized the coupling and uncoupling of brain interactions during different visuospatial WM processes, especially in situations where attention must be flexibly engaged to relevant information while handling distractions. Indeed, the ability to store information in WM and resist distractions can be impaired in PD (Fallon, Mattiesing, Muhammed, Manohar, & Husain, 2017; Lee, Cowan, Vogel, Rolan, & Valle‐Inc, 2010).

Working memory capacity depends on selectively encoding relevant information and ignoring irrelevant information, which needlessly consumes capacity (Vogel, McCollough, & Machizawa, 2005). It is governed by several integrated regions of the prefrontal cortices (Christophel, Klink, Spitzer, Roelfsema, & Haynes, 2017; Ichihara‐Takeda & Funahashi, 2007; Linden et al., 2003; McNab et al., 2008; Murty et al., 2011), striatum, thalamus (Cools & D'Esposito, 2011; McNab & Klingberg, 2008; Murty et al., 2011), and midbrain dopaminergic nuclei (Cools & D'Esposito, 2011). Neurocomputational models of WM propose that oscillatory activity in recurrent prefrontal cortex circuits maintain goal‐relevant information, whereas the basal ganglia and thalamus act as a gate to flexibly select and update the contents of WM and prevent irrelevant information from being stored (Hazy, Frank, & O'Reilly, 2007). Thus, the striatal‐thalamo‐prefrontal cortical network sets the system to selectively attend (McNab & Klingberg, 2008). A growing body of research also emphasizes independent contributions of parietal‐occipital areas in WM storage (Galeano Weber, Hahn, Hilger, & Fiebach, 2017; Murray, Jaramillo, & Wang, 2017) and their bidirectional connections with prefrontal cortex (Johnson et al., 2017), which may be lost in PD (Trujillo et al., 2015).

Although altered WM in PD is often attributed to frontostriatal dysfunction, increases, decreases, or no change in frontal and/or striatal activation have been reported (Caminiti, Siri, Guidi, Antonini, & Perani, 2015; Ekman et al., 2012; Lewis, Dove, Robbins, Barker, & Owen, 2003; Marklund et al., 2009; Mattay et al., 2002; Poston et al., 2016; Simioni, Dagher, & Fellows, 2017; Trujillo et al., 2015). Discrepant findings may relate to small PD samples (*n* ≤ 19) (Caminiti et al., 2015; Lewis et al., 2003; Mattay et al., 2002; Simioni et al., 2017; Trujillo et al., 2015), the use of diverse tasks (e.g., n‐back; Sternberg) that emphasize disparate processes, and differences in patients' medication status (on, off, and de novo). Few studies have rigorously screened PD patients for mild cognitive impairment (MCI) (Poston et al., 2016), such that neurodegenerative changes before cognitive symptoms manifest are poorly understood. Scant attention has also been given to neurocognitive mechanisms that mediate different facets of WM, such as memory encoding, retrieval and distraction resistance, which may be more or less vulnerable in PD (Fallon, Mattiesing, et al., 2017; Lee et al., 2010; Pillon, Deweer, Agid, & Dubois, 1993). Notably, much of what we know about abnormal brain functioning during WM comes from comparisons between PD and control groups in regional activation (Mattay et al., 2002; Poston et al., 2016), which are insensitive to abnormal communications of WM hubs with other brain regions.

To address these gaps, the present study characterized disturbances in the context‐dependent functional connectivity of regions or hubs that normally modulate WM. Cognitively normal PD and control participants underwent fMRI while performing a visuospatial WM task. To identify sources of brain dysfunction, distraction and memory‐load modulated connectivity were studied for encoding and retrieval phases of the task. Owing to aberrant frontostriatal activation in PD during WM (Lewis et al., 2003; Marklund et al., 2009; Poston et al., 2016), we hypothesized that during encoding, context‐dependent connectivity of regions within the striatal‐thalamocortical circuit would be abnormal in PD during distraction (Ekman, Fiebach, Melzer, Tittgemeyer, & Derrfuss, 2016; McNab & Klingberg, 2008) and higher memory loads, which impose a greater burden on WM (Hazy et al., 2007; Nee & Brown, 2013). If dopamine depletion in PD disrupts the fidelity by which information is encoded into WM (Cools & D'Esposito, 2011), we predicted that during retrieval both distraction‐ and load‐dependent connectivity would be altered in the superior parietal cortex, which represents the contents of visual WM (Christophel, Cichy, Hebart, & Haynes, 2015; Galeano Weber, Peters, Hahn, Bledowski, & Fiebach, 2016). We also hypothesized that aberrant connectivity features would predict individual differences in memory capacity and distraction resistance.

## 2 METHODS

### 2.1 Participants

The sample consisted of 30 cognitively normal PD participants who met the PD United Kingdom Brain Bank Criteria and 30 healthy controls. Exclusion criteria included metal in the head, neurological diagnoses other than PD, psychiatric diagnoses, history of alcohol or substance abuse, positive MRI findings (e.g., infarcts, vascular disease), use of anticholinergics or cognitive medications (e.g., Donepezil), color vision deficiency, and complaints of cognitive deficits. PD volunteers with tremors that might cause head motion were also excluded. PD and control volunteers were excluded if they met the Movement Disorders Society Level II criteria for PD‐MCI (Litvan et al., 2012). MCI was defined as >1.5 *SD* below the control group mean on at least two tests in a single domain or different domains. PD volunteers were also excluded if they reported problems with cognitive functioning in daily life (UPDRS Part I, item 1). PD participants were taking dopamine agonist monotherapy (*n* = 3), levodopa monotherapy (*n* = 7), or levodopa combination therapy (*n* = 20), and were in Hoehn and Yahr Stages 1 (*n* = 3), 2 (*n* = 15), and 3 (*n* = 12). Neuropsychological testing was conducted when patients were on medication. For MRI scanning, patients were off medication for a minimum of 14 hours. The Unified Parkinson's Disease Rating Scale (UPDRS) Part III total motor score was significantly greater off than on medications (Table 1). The Institutional Review Board at the VA San Diego Healthcare System approved the study. All subjects signed written informed consent.

**Table 1:** Demographic, clinical and cognitive variables

	Parkinson's	Control	*p*	η_p_^2^
Age (years)	67.6 (7.5)	68.6 (7.2)	.59	0.01
Education (years)	17.1 (2.3)	16.5 (1.8)	.32	0.02
Sex (% females)	27.0	63.0	.01	
Handedness (% right‐handed)	90.0	90.0	.55	
Wechsler test of adult Reading	44.6 (4.9)	45.2 (4.1)	.47	0.01
Mini‐mental status exam	29.3 (0.9)	29.5 (0.7)	.35	0.02
Hamilton depression scale	3.5 (2.4)	2.0 (2.6)	.06	0.07
Epworth sleepiness scale	8.8 (4.0)	7.0 (2.7)	.08	0.06
Disease duration (years)	5.4 (3.9)			
Levodopa dosage equivalence^a^	736.9 (400.4)			
UPDRS part III on^b^	27.7 (13.3)			
UPDRS part III off	37.5 (14.5)			
**Attention and working memory**				
Adaptive digit ordering (maximum span)	5.5 (1.1)	5.6 (1.3)	.69	0.00
Attention subscale (MDRS)	36.1 (1.2)	36.2 (1.0)	.93	0.00
**Executive (DKEFS)**				
Letter fluency	38.2 (11.3)	46.3 (14.01)	.02	0.09
Inhibition/switching	67.6 (19.7)	63.3 (16.7)	.46	0.01
**Memory**				
CVLT‐II short delay free recall	8.9 (2.9)	10.9 (2.9)	.08	0.06
CVLT‐II long delay free recall	9.5 (3.1)	11.5 (2.9)	.05	0.07
Logical memory II (WMS‐III)	29.4 (5.8)	31.2 (8.6)	.53	0.01
**Visuospatial**				
Judgment of line orientation	24.7 (4.5)	24.8 (3.3)	.49	0.01
Hooper visual organization	25.6 (2.3)	25.8 (2.9)	.37	0.02
Benton visual form discrimination^c^	30.6 (1.8)	29.2 (3.5)	.10	0.05
**Language**				
Boston naming	57.9 (2.0)	57.2 (2.4)	.46	0.01
Similarities (WAIS‐IV)	28.5 (4.1)	28.4 (5.6)	.77	0.00

*Note*: Tabled values are unadjusted raw score means (*SD*). Group differences were tested using ANCOVA (sex adjusted) and chi‐square statistics.

Abbreviations: CVLT‐II, California Verbal Learning Test Version 2; DKEFS, Delis Kaplan Executive Function System; MDRS, Mattis Dementia Rating Scale; WMS‐III, Wechsler Memory Scale Version 3.

a Unified Parkinson's Disease Rating Scale (UPDRS) Part III motor scores were significantly greater off than on medications (F = 113.6, *p* < .00001).

b Levodopa dosage equivalence was calculated using the method of Tomlinson et al., 2010.

c There was missing data on the Benton Visual Form Discrimination test for five control and four PD participants.

The groups did not differ in age, educational level, and premorbid intelligence (Wechsler Test of Adult Reading), but the control group contained a higher percentage of females (Table 1). Analyses of covariance (ANCOVA), adjusting for sex, showed that the PD group had significantly lowered scores than controls on the Letter Fluency test and showed a trend (*p* = .05) for lower scores on long delay free recall of the California Verbal Learning Test (CVLT). While this indicates a decline in verbal fluency and a trend for episodic memory decline at the group level, individual patients did not exhibit clinically significant cognitive decline indicative of MCI. Self‐reports of daytime sleepiness (Epworth Sleepiness Scale) and depression symptoms (Hamilton Depression Scale) did not differ between the groups. Depression symptoms in both groups were within the normal to mild range (0 to 8).

### 2.2 Working memory paradigm

**Figure 1:**
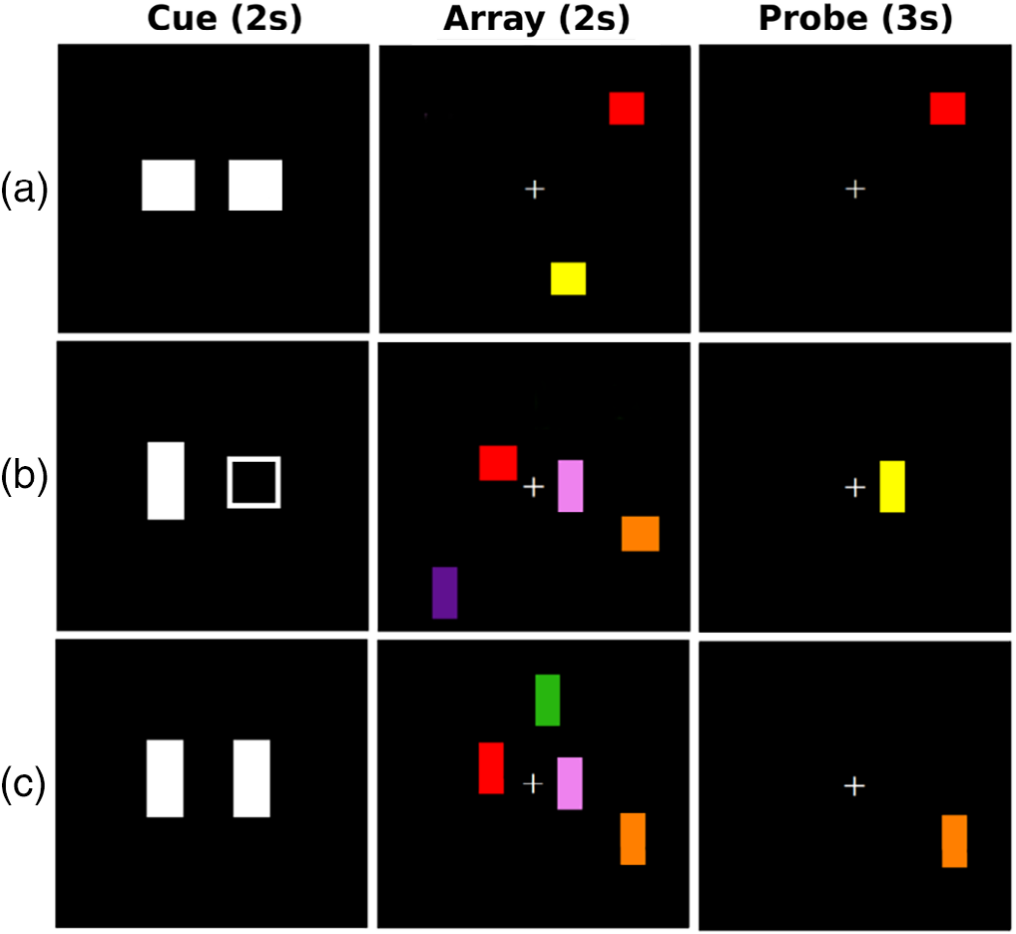
Illustration of the visuospatial working memory paradigm. A trial began with a cue, which signaled the shape to attend to. On no distraction trials (1a and 1c), the cue was either two filled squares or two filled rectangles that designated the to‐be‐attended shape. On distraction trials (1b), a rectangle and square were presented, instructing the subject to attend to the filled shape and ignore the unfilled shape. After a random delay (2,000 to 2,900 ms), one of three array types was presented for 2 s (*encoding phase*): two color shapes, no distractor (1a); 2) four color shapes, no distractor (1c); and two color shapes, two distractor shapes (1b). After a random delay period (2,000 to 3,300 ms), a probe was presented (*retrieval phase*) in a location occupied by one of the attended shapes in the array. The participant decided if the probe was the same or different color than the shape in the same location of the array by pressing their right index or middle finger, respectively

The task was a modified version of similar paradigms (Vogel et al., 2005), and manipulated visuospatial WM load and distraction (Figure 1). On each trial, a 2 s cue was presented, signaling to which shape to attend. On no distraction trials (Figure 1a,1c), the cue was either two filled squares or rectangles that designated the to‐be‐attended shape. On distraction trials (Figure 1b), a rectangle and square were presented, instructing the subject to attend to the filled shape and ignore the unfilled shape. After a jittered delay (2000 to 2,900 ms), one of three array types was presented (*encoding phase*): (a) two color shapes, no distractors; (b) four color shapes, no distractors; and (c) two color shapes, two distractor shapes. The array offset was followed by a jittered delay period (2,000 to 3,300 ms) and then a single probe was presented (*retrieval phase*) in a location occupied by one of the shapes in the array. The participant decided if the probe was the same or different color than the shape in the same location of the array by pressing their right index or middle finger. Thus, memory was required for both location and color. The intertrial interval (time from offset of the probe to onset of the cue) was jittered between 4 and 5.3 s. Randomized stimulus timing parameters were optimized using RSFgen from the Analysis of Functional NeuroImages (AFNI) software (Cox, Chen, Glen, Reynolds, & Taylor, 2017a). We assumed that the encoding phase included storage and maintenance processes, whereas the retrieval phase involved processes that gain access to stored contents.

There were 24 trials per array type (50% same and 50% different probes) for a total of 72 trials. Array types were randomly presented across two blocks of 36 trials each. To probe for brain circuits that modulate memory load, arrays of four and two shapes without distraction were compared. To probe for brain circuits that modulate filtering, arrays containing two shapes with or without two distractor shapes were compared. The manipulations of load and distraction were based on pilot testing showing that they produced large effect sizes on performance, while maintaining accuracy at or above 75%, which was essential for obtaining an adequate number of correct trials during fMRI. The dependent measures were (a) percent correct, (b) d prime (d′), (c) memory capacity (K = set size * [hits‐false alarms]), and mean reaction time (RT) for correct trials (time from onset of probe to key press).

### 2.3 Imaging protocols

Imaging was conducted on a GE MR750 Discovery 3 Tesla system with an eight‐channel head coil. Head motion was limited by foam pads inserted between the head and the coil. Visual stimuli were viewed through a NordicNeuroLab goggle system. Non‐ferrous key pad devices interfaced with a computer recorded task performance during fMRI for off‐line analysis.

High‐resolution T1‐weighted anatomical images were acquired that maximized differentiation of the white and gray matter boundary (3D spoiled gradient‐recalled at steady state, minimum full TE, 7.8 ms TR, 600 TI, 8° flip angle, 1‐mm slices, 25.6 cm FOV). For task‐activated fMRI, echo‐planar images (EPI) were acquired in an oblique orientation (perpendicular to the anterior–posterior commissure) to minimize susceptibility artifacts, using a single‐shot, blipped, gradient‐echo, EPI pulse sequence (30.5 ms TE, 2.0 s TR, 90° flip angle, 25.6 cm FOV, 64 × 64 matrix, 37 contiguous 4 mm slices (3.75 × 3.75 × 4 mm voxel size) that provided coverage of the entire brain.

### 2.4 Image analyses

Data were analyzed using the AFNI software (Cox, Chen, Glen, Reynolds, & Taylor, 2017b). After discarding the first four volumes of the time series, functional data were motion corrected using SLice‐Oriented MOtion Correction (Beall & Lowe, 2014), which performs an in‐plane slice‐wise motion registration, followed by an out‐of‐plane motion parameter estimation and regularization. Before motion correction, the groups did not differ in maximum scan‐to‐scan displacement (Control: 0.33 (*SD* = 0.18); PD: 0.37 (*SD* = 0.22); F < 1.0, *p* = .49, η_p_^2^ = 0.01) or framewise displacement (Control: 0.26 (*SD* = 0.12); PD: 0.27 (*SD* = 0.13; F < 1.0, *p* = .61, η_p_^2^ = 0.01). Thus, procedures to restrict head motion were highly effective. Motion correction further reduced small fluctuations in head motion to less than 0.06 mm (maximum displacement: Control: 0.03 (*SD* = .02); PD: 0.03 (*SD* = 0.02); F < 1.0, *p* = .82, η_p_^2^ = 0.001) and 0.05 mm (framewise displacement: Control: 0.02 (*SD* = 0.01); PD: 0.02 (*SD* = 0.02); F < 1.0, *p* = .84, η_p_^2^ = 0.001) in all subjects. Motion regressors were therefore not included in the voxelwise or gPPI statistical models. After motion correction, the volumes were time shifted, transformed to Talairach space, and spatially filtered (6 mm Gaussian kernel).

#### 2.4.1 Voxelwise tests of condition effects

First‐level analyses tested for the effect of the task conditions on brain activation in pooled analysis of both PD and control participants, which were conducted separately for the encoding and retrieval phases of the task. AFNI 3dDeconvolve was used to estimate the hemodynamic response function (HRF) of each voxel using multiple linear regressions. The analysis pipeline included deconvolution of each subject's time series for correct trials of each condition (two shapes, no distractors; four shapes, no distractors; two shapes, two distractors). Each HRF was estimated relative to the baseline state (filler images). Incorrect trials were regressed out of the time series at each voxel. For each contrast of interest, the control condition was two shapes, no distractors. Contrasts of interest compared the differences in the magnitude of the signal (beta coefficient) during the encoding and retrieval phases for: (a) four shapes, no distractors minus the control condition (load effect) and (b) two shapes, two distractors minus the control condition (distraction effect).

The effects of the task conditions on brain activation were tested in the entire sample using 3dMVM (Chen, Adleman, Saad, Leibenluft, & Cox, 2014). Monte Carlo simulations with 5,000 iterations (3dClustSim using the ACF method) to compute the voxel‐probability and minimum cluster‐size threshold needed to obtain a familywise alpha (Cox et al., 2017b). Because spatial thresholds are biased against small activation clusters such as striatum and hippocampus/parahippocampus, which were regions of interest (ROI), thresholds were derived separately for these structures and cortical volumes. A corrected alpha of *p* < .05 was obtained using a voxelwise probability of *p* < .0001 and a minimum cluster size of ≥38 voxels for the cortex and *p* < .01 and a minimum cluster size of >20 voxels for the striatum and hippocampus/parahippocampus.

#### 2.4.2 Voxelwise tests of group differences in task conditions

To test for group differences in regions that showed the above condition effects, second‐level ANCOVAs (sex adjusted) tested for the effects of group, group by distraction, and group by memory load for regions showing significant condition effects during the encoding and retrieval phases. The *p* values for group tests were adjusted for multiple comparisons using the false discovery rate (FDR) method (q < .05), separately for each phase.

#### 2.4.3 Context‐dependent connectivity analyses

The hypotheses focused on testing whether group differences in the context‐dependent connectivity of a seed ROI with other brain regions depended on memory load and the presence of distraction. The generalized psychophysical interaction (gPPI) model as implemented in AFNI was used. The gPPI approach explores the physiological response (i.e., hemodynamic response convolved blood‐oxygen‐level dependent signal) of a ROI in terms of its context‐dependent response with other regions (Cisler, Bush, & Steele, 2014; McLaren, Ries, Xu, & Johnson, 2012). This yields measures of task‐modulated connectivity between two or more regions. The selection of ROI or seeds for the gPPI analyses was empirically driven. We identified ROI from the first‐level voxelwise analyses that showed significant effects of distraction and load for each phase (encoding and retrieval) of the analyses in the combined PD and control groups (Tables S1 and S2). To reduce the dimensionality of the data set and minimize the number of multiple comparisons, seeds were constrained to regions commonly implicated in WM, namely frontal–parietal (Linden et al., 2003; McNab & Klingberg, 2008; Murray et al., 2017), occipital (Galeano Weber et al., 2017), parahippocampus (Ranganath & Ritchey, 2012), basal‐ganglia (Cools & D'Esposito, 2011; McNab & Klingberg, 2008; Murty et al., 2011), and thalamus (Hazy et al., 2007; Schmitt et al., 2017). Table S3 describes the seeds that were constructed from ROI from the first‐level voxelwise tests. From the voxelwise tests of distraction (Table S1), 10 and 6 ROI were identified as key WM regions for the encoding and retrieval phases, respectively. From the voxelwise tests of memory load (Table S2), 11 and 10 ROI were identified as key WM regions for the encoding and retrieval phases, respectively.

To define regions for the gPPI analyses, 7 mm diameter seeds were placed in the vicinity of peak activation in ROI that showed an effect of load or distraction for the encoding or retrieval phase. The physiological variable was created by extracting the mean deconvolved time courses from a seed region for each individual. The PPI interaction terms were computed as the cross product of the physiological variable and each of the six task conditions (i.e., two shapes no distraction, two shapes distraction, and four shapes no distraction for the encoding and retrieval phases). Nuisance variables were error trials for each of the six task conditions). This resulted in a first‐level model (i.e., one per seed) with six nuisance variables and 13 regressors (i.e., six task conditions, six PPI interaction terms and the time course of one seed). The regression produced correlation maps for the time course in the seed ROI with the time course from all other brain voxels as a function of a task condition and phase of the task. Fisher z transforms were applied to the correlation maps. The focus of the second‐level analyses was the interaction of group (PD and controls) with the contrasts of interest from the first level analyses (i.e., distraction and load effects), as implemented using (AFNI 3dMVM). The analyses were thresholded using a voxelwise‐probability of *p* < .005 and minimum cluster size of 41 voxels (5,000 simulations using the AFNI ACF method). To further adjust for the analysis of multiple seeds, the FDR adjustment (q < .05) was applied to the uncorrected p values from these analyses.

### 2.5 Statistical analyses

Owing to group differences in sex, analyses were conducted on sex‐adjusted standardized residuals computed for cognitive (WM measures, neuropsychological raw test scores) and gPPI connectivity variables that differed between the groups, which included context‐dependent connectivity features that were stronger and weaker in the PD group relative to controls. The FDR was used to correct for multiple analyses (q < .05), except where noted.

#### 2.5.1 Relationships between context‐dependent connectivity and WM proficiency

Using stepwise multiple regression sets of functional connectivity variables that differed between the groups (independent variables) were regressed onto key WM measures (dependent variable), separately for each phase of the task. This analytic approach identified connectivity variable(s) that best accounted for individual differences in memory capacity and distractor resistance. Memory capacity (K) for the large array (four shapes no distractors) was used as it best reflects the upper limit of capacity. To measure distraction resistance, d′ and RT for two shapes no distractors was subtracted from d′ and RT for two shapes with distractors. Regression analyses were conducted separately for the PD and control groups. Owing to a priori hypotheses regarding these associations and their exploratory nature, *p* values were uncorrected and should be cautiously interpreted.

#### 2.5.2 Context‐dependent connectivity associations with neuropsychological variables

Using stepwise multiple regressions, sets of functional connectivity variables (independent variables) were regressed onto selected neuropsychological test performances in three cognitive domains most closely related to visuospatial WM task demands: (a) executive functioning (DKEFS Inhibition and Switching subtest), (b) short‐term episodic memory (CVLT short delay free recall), (c) complex visual form discrimination (Benton Visual Form Discrimination; BVFD), and (d) visual organization (Hooper Visual Organization; HVO). For each neuropsychological test, FDR adjustments (q < .05) used the number regions that showed group differences across the two phases of the task.

#### 2.5.3 Sensitivity of context‐dependent connectivity features

Discriminant function analyses were performed on connectivity variables or features that significantly differed between the groups to characterize their sensitivity and specificity, which could inform the refinement of measures that may serve as markers of WM dysfunction in future longitudinal studies. To reliably estimate classification accuracy, a bias‐corrected and accelerated bootstrap (1,000 bootstrapped samples) method was used. Receiver operating curve analyses (ROC) then evaluated the goodness‐of‐fit of the discriminant model by analyzing the area under the curve (AUC) for the sensitivity and specificity distributions relative to the null hypothesis (AUC = 0.50). The AUC indicates the overall accuracy of a linear weighted‐combination of variables in distinguishing a PD patient from controls. These analyses were conducted separately for aberrant distraction‐ and load‐dependent connectivity variables.

## 3 RESULTS

### 3.1 Behavioral results

**Figure 2:**
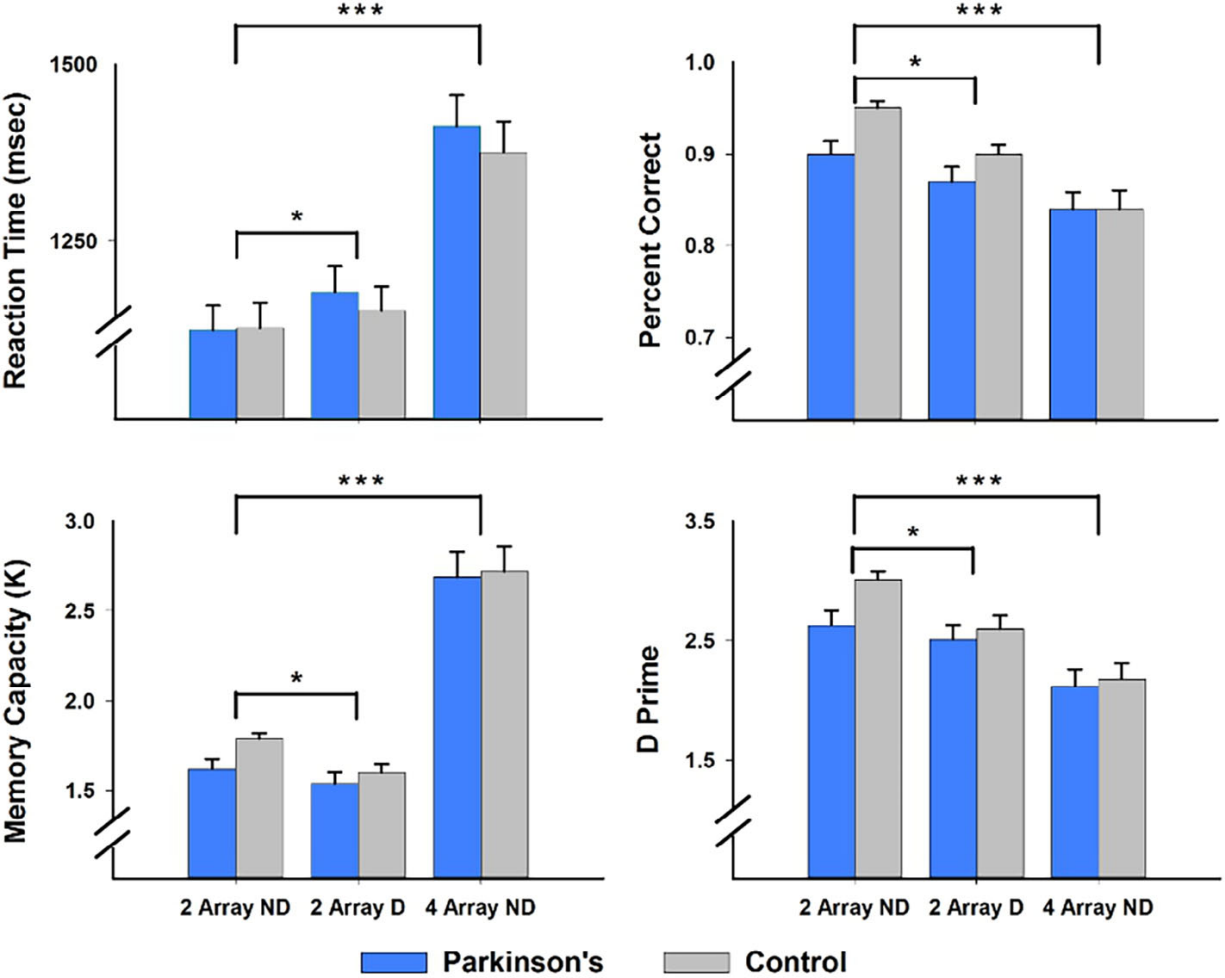
Working memory performance in the PD and control groups. Mean and standard error bars are graphed for reaction time, percent correct, memory capacity (K), and d prime. Group differences in the effects of distraction and memory load were not significant for any of the working memory measures. Brackets signify the significance of distraction and memory load effects on working memory performance in both groups (***p < .0001; *p < .01)

ANCOVA tested for group differences in the WM measures separately for the analyses of memory load and distraction effects. Sex and its interactions with load and distraction were not significantly related to any of the behavioral variables (Figure 2). Memory load and distraction had significant effects on all behavioral variables. In comparison to small array sizes, large memory arrays were associated with longer RTs (F = 239.9, *p* < 2.9E−22, η_p_^2^ = 0.81), lower accuracy (F = 48.2, *p* < 3.6E−9, η_p_^2^ = 0.45), lower d′ (F = 47.9, *p* < 4.0E−9, η_p_^2^ = 0.45), and higher K (F = 109, *p* < 6.1E−15, η_p_^2^ = 0.65). Group differences and group interactions with array size were not significant. Relative to the no distraction condition, distraction was associated with longer RTs (F = 7.6, *p* < .01, η_p_^2^ = 0.12), lower accuracy (F = 8.5, *p* < .01, η_p_^2^ = 0.13), and lower d′ (F = 8.4, *p* < .01, η_p_^2^ = 0.13), and lower K (F = 8.5, *p* < .006, η_p_^2^ = 0.13). Group differences and group interactions with distraction condition were not significant. Measures of WM performance were not significantly correlated with disease duration, levodopa dosage equivalence, or motor symptom severity scores (UPDRS Part III on and off medication) (*p* > .10).

### 3.2 Voxelwise test results

**Figure 3:**
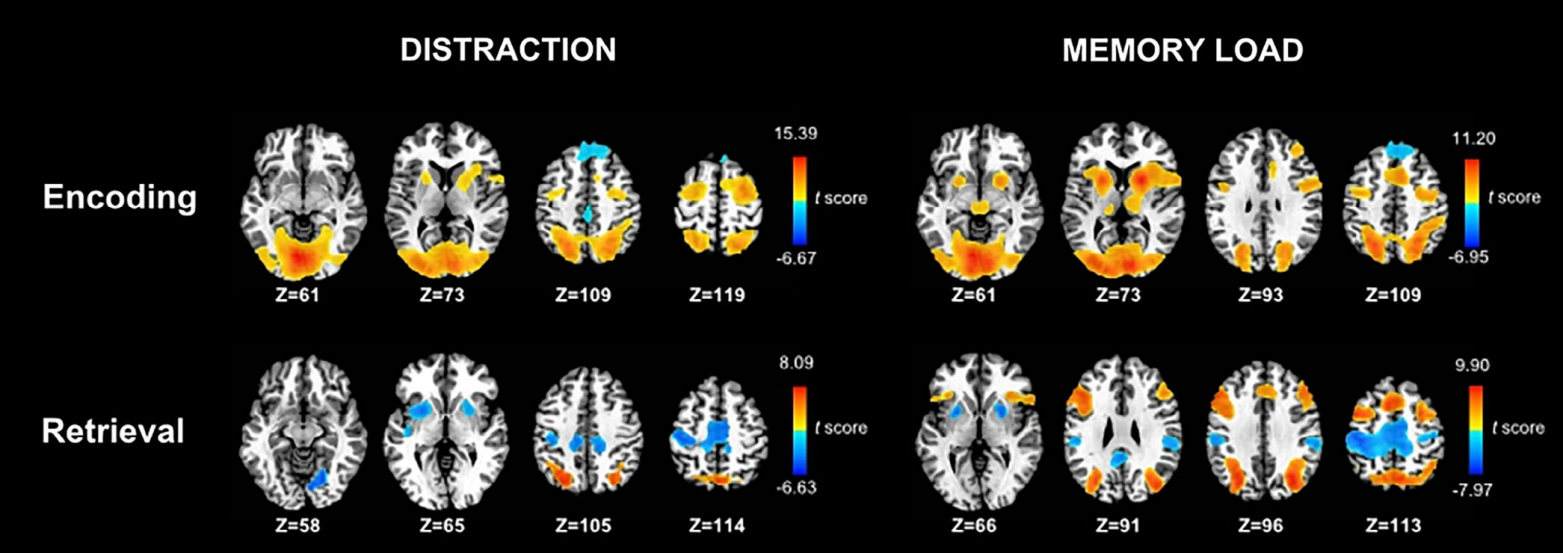
Effects of distraction and memory load on voxelwise tests of brain activation during the storage and retrieval phases of the working memory task. Analyses were conducted on the combined PD and control samples. The left side displays regional activations that were greater for distraction than no distraction conditions (warm colors) and greater for no distraction than distraction conditions (cool colors). The right side displays regional activations that were greater for large (*n* = 4) than small (*n* = 2) arrays (warm colors) and greater for small than large arrays (cool colors). Color bars display the range of t scores for the significant condition effects. Tables S1 and S2 describe the characteristics of regional activations associated with the voxelwise tests of condition effects

First‐level analyses tested for the effects of distraction and memory‐load on brain activation during the encoding and retrieval phases of the task to identify ROI or seeds for subsequent gPPI analyses (voxel‐probability and minimum cluster‐size thresholded). Figure 3 displays the results from these analyses. Details of the regions that showed significant distraction and memory load effects are given in Tables S1 and S2, respectively, which also summarize the results from tests of group differences in these regions.

#### 3.2.1 Distraction effects

During encoding, activation was greater in the distraction than no distraction condition for the superior parietal, precuneus, and occipital cortices, cerebellum, middle frontal gyrus (BA 6), the posterior parahippocampal cortex (PHC), putamen, and globus pallidus (GP). In contrast, activation was greater in the no distraction than the distraction condition for the anterior PHC and superior frontal gyrus (BA 8, 9). For retrieval, activation was greater in the distraction than no distraction condition for the superior parietal cortex and precuneus, whereas activation was greater in the no distraction than distraction condition for the supplementary motor area (SMA), sensorimotor cortex, lingual gyrus, putamen, and GP.

#### 3.2.2 Load effects

During encoding, activation in almost all regions (prefrontal, dorsolateral prefrontal cortex [DLPFC], superior parietal, precuneus, occipital, PHC, basal ganglia) was greater for the larger than the smaller array size, except for the superior frontal gyrus. During retrieval, activation in precuneus and inferior parietal cortex, inferior and middle frontal gyri, DLPFC, and pre‐supplementary motor area (preSMA) was greater for the larger than the smaller array size. In contrast, motor circuit (putamen, GP, SMA proper, cingulate, precentral gyrus), posterior cingulate, and inferior parietal cortex activations were greater for the smaller than larger arrays.

#### 3.2.3 Group differences

ANCOVAs tested for group differences in regions that showed significant effects of distraction and memory load in the above analyses. Inclusion of sex as a covariate did not alter the significance of group or group by condition effects. Group interactions with distraction and load were not significant for any regions. During retrieval, precuneus, superior parietal cortex, and SMA activations were greater in the control than the PD group (FDR adjusted), regardless of the distraction condition (Table S1). Group differences in memory load effects were not significant for any regions (FDR adjusted) (Table S2).

### 3.3 Context‐dependent functional connectivity (gPPI) results

**Figure 4:**
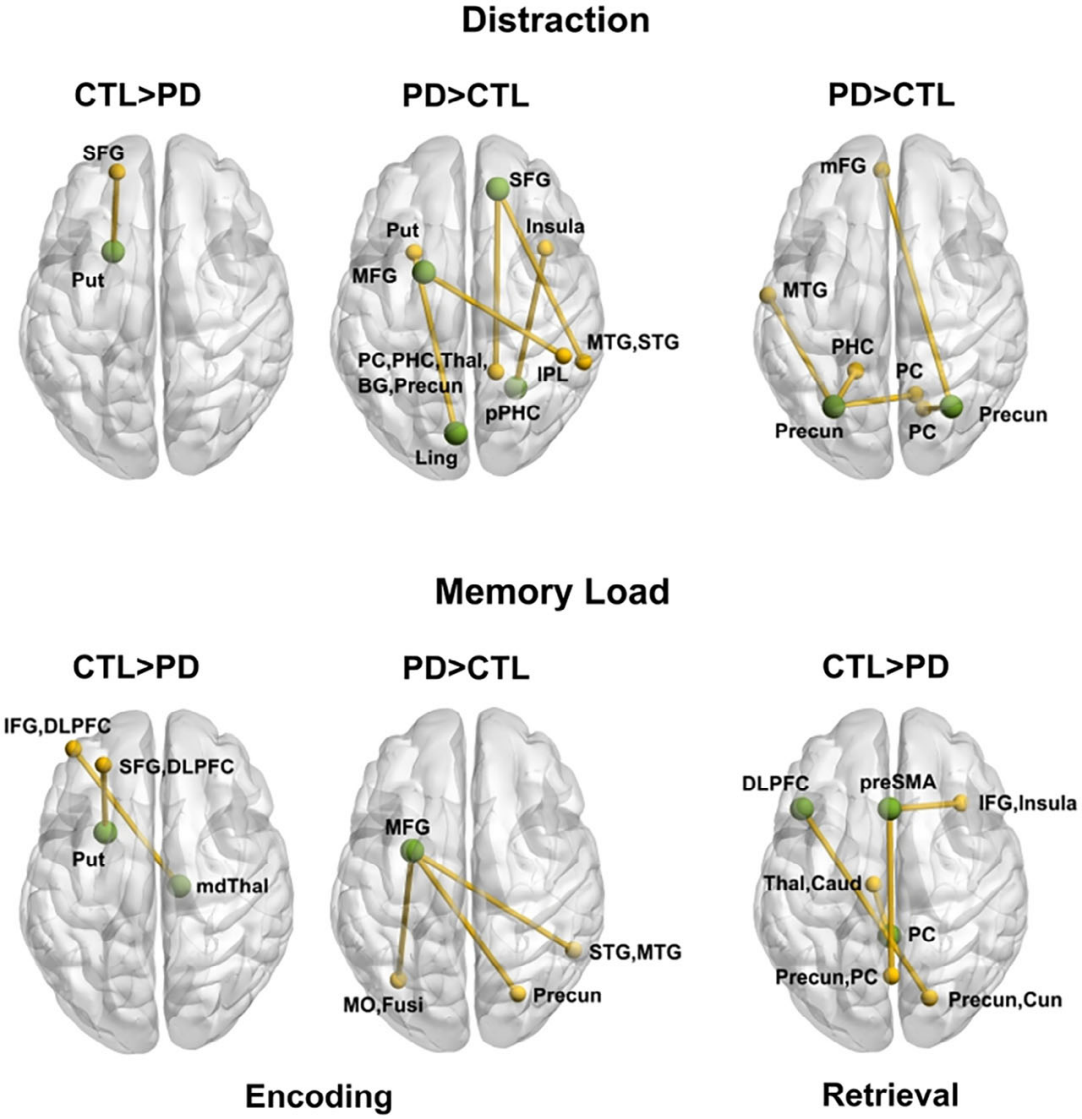
Group differences in context‐dependent functional connectivity during working memory. The figure illustrates significant group differences in the connectivity of a seed ROI (green circles) with other brain regions (gold circles and lines) during the encoding and retrieval phases of the task. *Top row*: Group differences in distraction‐dependent connectivity. *Bottom row*: Group differences in memory load‐dependent connectivity. Tables 2 and 3 provide the characteristics of distraction and load‐related connectivity features that differed between groups. BG, basal ganglia; DLPFC, dorsal lateral prefrontal cortex; Fusi, fusiform gyrus; IFG, inferior frontal gyrus; IPL, inferior parietal lobule; mdThal, mediodorsal thalamus; mFG, medial frontal gyrus (BA 10); MFG, middle frontal gyrus (BA 6); MO, middle occipital (BA 19); MTG, middle temporal gyrus; PC, posterior cingulate; precun, precuneus; PHC, parahippocampal cortex; pPHC, posterior parahippocampal cortex; SFG, superior frontal gyrus (BA 8); preSMA, presupplementary motor area; STG, superior temporal gyrus; Thal, thalamus

Figure 4 illustrates the significant group differences in the connectivity of a seed (green circles) with other brain regions (gold circles and lines) during each phase of the task. Inclusion of sex as a regressor did not alter the outcome of most results. Group differences in distraction‐ and load‐dependent connectivity are displayed at the top and bottom of the figure. Tables 2 and 3 detail the features that showed group differences in context‐dependent connectivity along with features for which group differences were not found, which are illustrated in Figure S1. Table S4 and Figure S1 show seeds for which context‐dependent connectivity were not found in either group. In the PD group, disease duration, levodopa dosage equivalence, and motor symptom severity (on and off medication) were not related to gPPI variables that showed abnormal context‐modulated connectivity.

**Table 2:** Group differences in distraction‐dependent connectivity during each phase of the task

Seed	Region	Voxels	X	Y	Z	*p* value	η_p_^2^
**Encoding phase: Distraction > no distraction**							
**PD > control**							
R SFG (BA 8)	R PC, PHC, mdThal, pulvinar, putamen, GP, precuneus	324	15	−48	8	.00006	.25
	R MTG, STG (BA 21,22)	149	59	−43	5	.00004	.26
L MFG (BA 6)	R IPL (BA 40)	43	49	−40	53	.0001	.24
R pPHC	R insula	51	36	14	−1	.001	.18
L lingual	L putamen	44	−24	10	−1	.0001	.23
**Control > PD**							
L putamen	L mSFG (BA 9)	51	−19	52	24	.0001	.24
							
**Encoding phase: No distraction > distraction**							
**Control = PD**							
R MFG (BA6)	L IPL (BA 40)	58	−56	−36	27	.68	.00
R precuneus	L caudate	41	−35	−30	26	.69	.00
L precuneus	L posterior insula	44	−35	−30	26	.58	.01
R putamen	L PC (BA 30, 31)	46	−16	−62	16	.50	.01
	B paracentral, cingulate (BA 5, 31)	44	2	−34	55	.88	.00
**Retrieval phase: Distraction > no distraction**							
**PD > control**							
L Precuneus	L MTG (BA 21)	50	−61	−10	−17	.00006	.25
	L PHC	42	−17	−48	−6	.0002	.22
	R PC	42	13	−64	11	.002	.16
R Precuneus	B mFG (BA 10)	72	−4	53	−5	.00006	.25
	R PC	60	17	−65	15	.003	.15

*Note*: X, Y, Z coordinates are from the MNI atlas. *p* values (uncorrected) and η_p_^2^ values are based on ANCOVA tests for the group by distraction interaction. All significant uncorrected *p* values remained significant after the FDR adjustment (q < .05).

Abbreviations: B, L, and R, bilateral, left, and right hemispheres; BA, Brodmann area; GP, globus pallidus; IPL, inferior parietal lobule; mdThal, mediodorsal thalamus; mFG, medial frontal gyrus; MFG, middle frontal gyrus; MTG, middle temporal gyrus; PC, posterior cingulate; pPHC, posterior parahippocampal cortex; mSFG, medial superior frontal gyrus; STG, superior temporal gyrus.

**Table 3:** Group differences in memory load‐dependent connectivity during each phase of the task

Seed	Region	Voxels	X	Y	Z	*p* value	η_p_^2^
**Encoding phase (4 > 2 arrays)**							
**Control > PD**							
L putamen	LSFG (BA 8), DLPFC (BA 9)	74	−26	43	35	.00002	.28
R mdThalamus	L IFG (BA 10), DLPFC (BA 46)	67	−41	51	6	.00003	.27
**PD > control**							
L MFG (BA 6)	R precuneus	148	26	−73	28	.0004	.20
	L MO, fusiform (BA 19,37)	146	−34	−66	−9	.0002	.22
	R STG, MTG (BA 21,22)	134	54	−51	3	.0002	.22
**Control = PD**							
R caudate	B anterior cingulate	41	2	46	−8	.62	.004
**Retrieval phase (4 > 2 arrays)**							
**Control > PD**							
L DLPFC (BA 9)	R precuneus, cuneus	90	20	−75	43	.00001	.29
B preSMA	B precuneus, PC	139	1	−64	37	.00004	.26
	R IFG, insula (BA 13,47)	68	36	23	−9	.00004	.26
B PC	L thalamus, caudate tail	95	−8	−18	15	.00001	.29
**Control = PD**							
L MFG BA6	L MOC (BA 19)	46	−41	−73	9	.73	.002
R precuneus	R PC	43	15	−43	17	.10	.048
R putamen	L cerebellar tonsil	60	−24	−55	−41	.59	.005
	L precuneus	50	−28	−66	41	.29	.02

*Note*: X, Y, Z coordinates are from the MNI atlas. *p* values (uncorrected) and η_p_^2^ values are based on ANCOVA tests for the group by memory load interaction. All significant uncorrected *p* values remained significant after the FDR adjustment (q < .05).

Abbreviations: B, L, and R, bilateral, left, and right hemispheres; BA, Brodmann area; DLPFC, dorsal lateral prefrontal cortex; IFG, inferior frontal gyrus; mdThalamus, mediodorsal thalamus; MFG, middle frontal gyrus; MTG, middle temporal gyrus; MOC, middle occipital; PC, posterior cingulate; SMA, supplementary motor area; STG, superior temporal gyrus.

#### 3.3.1 Distraction‐dependent connectivity

Distraction‐related connectivity of WM hubs differed between the groups during the encoding and retrieval phases (Table 2). Stronger connectivity during distraction than no distraction was striking in the PD group. In the encoding phase, this included connectivity of bilateral frontal areas with regions of the ventral attention network (temporal, middle occipital), a memory encoding and retrieval system (precuneus, posterior cingulate, PHC), and subcortical regions (putamen, GP, thalamus). Connectivity of the posterior PHC and lingual gyrus with the salience network (insula) and the putamen was also stronger during distraction in the PD group. In the control group, these regions showed the opposite connectivity pattern (weaker connectivity during distraction than no distraction). In the control group, only connectivity of the left putamen with the superior frontal gyrus was stronger when encoding displays with distractors, whereas the PD group showed the opposite pattern of coupling (weaker connectivity during distraction). In contrast, no group differences were found for regions exhibiting stronger connectivity during no distraction than distraction (right middle frontal, bilateral precuneus, and right putamen) (Figure S1).

During retrieval, the PD group showed strengthened bilateral precuneus connectivity with a retrieval circuit (PHC, posterior cingulate) and a region of the ventral attention system (middle temporal) for targets that were encoded during distraction. The control group showed the opposite pattern of coupling.

#### 3.3.2 Load‐dependent connectivity

In both phases, load‐related connectivity also differed between the groups (Table 3). During encoding, stronger connectivity of striatal‐thalamocortical regions for large than small arrays was notable in the control group. Specifically, putamen and medial thalamus coupling was strengthened with superior frontal, DLPFC, and inferior frontal cortices. The PD group showed the opposite pattern of connectivity, failing to increase striatal‐thalamocortical connectivity when encoding large arrays. Rather, in the PD group left middle frontal connectivity was strengthened with the right precuneus and ventral attention regions (occipital, fusiform gyrus, and temporal cortices) when encoding large arrays. On the other hand, right caudate‐anterior cingulate connectivity was stronger for large than small arrays, but to the same extent in both groups.

During retrieval, only the control group showed stronger connectivity of the (a) left DLPFC with the right precuneus; (b) the preSMA with an encoding/retrieval system (precuneus and posterior cingulate) and the salience network (inferior frontal and insula), and (c) the posterior cingulate with thalamus/caudate tail. The PD group failed to increase connectivity of these regions when making a decision about a target encoded in a large array. In contrast, left middle frontal, right precuneus, and right putamen connectivity with ventral attention regions (occipital) and an encoding and retrieval system (precuneus, posterior cingulate) increased when retrieving a target encoded in a large array, but to the same extent in both groups (Figure S1).

### 3.4 Connectivity predictors of WM performance

**Figure 5:**
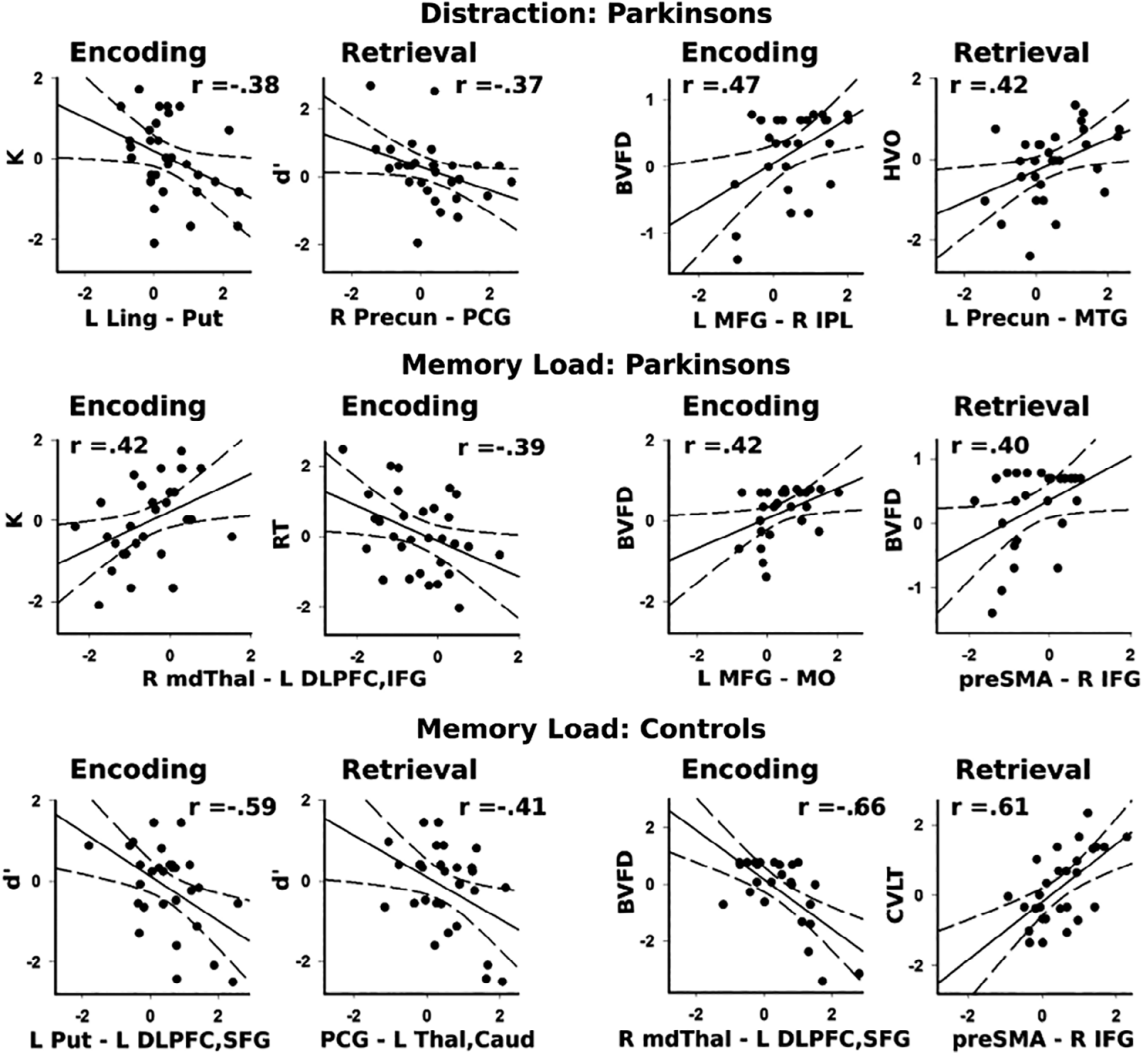
Relationship between context‐dependent connectivity and cognition. The graphs plot aberrant features of distraction and memory‐load dependent connectivity for encoding and retrieval phases and their correlations with visuospatial working memory (left columns) and neuropsychological test performances (right columns). Graphs are displayed for PD (top two rows) and control (bottom row) participants. Standardized residuals (sex adjusted) are plotted for all variables. Higher K values signify greater working memory capacity. More negative d′ values indicate less distraction resistance. More negative RTs indicate better distraction resistance. BVFD, Benton Form Discrimination test; CVLT, California Verbal Learning test (short delayed recall); HVO, Hooper Visual Organization test; L/R, left and right hemisphere; Caud, caudate; DPFC, dorsolateral prefrontal cortex; IFG, inferior frontal gyrus; IPL, inferior parietal lobule; Ling, lingual gyrus; mdThal, mediodorsal thalamus; MFG, middle frontal gyrus (BA 6); MTG, middle temporal gyrus; preSMA, presupplementary motor area; PCG, posterior cingulate gyrus; Precun, precuneus; Put, putamen; SFG, superior frontal gyrus; Thal, thalamus

Stepwise multiple regression analyses were conducted on K, d′ (distraction–no distraction), and RT (distraction–no distraction) to identify the best connectivity predictor(s) of WM capacity and distraction resistance, separately for the PD and control groups. The analyses were confined to connectivity predictors that differed between the groups, owing to our focus on abnormal context‐dependent connectivity in PD. Figure 5 (left two columns) displays plots of significant correlations between connectivity features and WM performance for each group.

#### 3.4.1 Distraction and WM performance

In the PD group, stronger distraction‐related connectivity was associated with lower K and less resistance to distraction (more negative d′ values). Specifically, during encoding, lower K values were associated with increased distraction‐related connectivity of the left lingual gyrus with left putamen (F = 4.64, *p* < .04, r = −.38). During retrieval, more negative d′ was associated with stronger right precuneus ‐ right posterior cingulate gyrus connectivity (F = 4.29, *p* < .05, r = −.37). In the control group, distraction‐related connectivity features were not significantly correlated with WM performance.

#### 3.4.2 Load and WM performance

In the PD group, stronger thalamus‐frontal cortex connectivity when encoding large arrays, which was a feature of connectivity in the control group (Table 3), was associated higher K (F = 5.85, *p* < .02, r = .42) and better resistance to distraction (more negative RTs) (F = 5.16, *p* < .03, r = −.39). Load‐related connectivity features during retrieval did not significantly correlate with WM measures.

In the control group, better WM was associated with stronger connectivity for a distinguishing feature of controls and weaker connectivity for a feature that characterized the PD group. During encoding, less resistance to distraction (more negative d′) was predicted by both stronger left putamen‐frontal cortex connectivity (r = −.59) and weaker left middle frontal‐occipital/fusiform connectivity (r = −.46) (F = 13.32, *p* < .0001, R^2^ = .57), the latter of which was a feature of stronger connectivity in the PD group (Table 3). During retrieval, more negative d′ was associated with stronger posterior cingulate‐thalamus/caudate connectivity for large arrays (F = 5.62, *p* < .025, r = −.41).

### 3.5 Connectivity predictors of neuropsychological test performances

Stepwise multiple regression analyses were conducted to identify predictor(s) of executive processing (DKEFS Inhibition and Switching), short‐term episodic memory (CVLT short delay free recall) and visuospatial processing (Benton Visual Form Test, Hooper Visual Organization). Figure 5 (right two columns) displays plots of significant correlations between connectivity features and neuropsychological test performance for each group.

#### 3.5.1 Distraction and neuropsychological performances

Distraction‐related connectivity variables did not predict executive processing or episodic memory in either group, but was related to visuospatial processing in the PD group. During encoding, stronger left middle frontal—left inferior parietal connectivity during distraction predicted better Benton Visual Form Test scores in the PD group (F = 6.66, q < .05, r = .47). During retrieval, stronger left precuneus—left middle temporal connectivity correlated with better Hooper Visual Organization scores in the PD group (F = 5.89, q < .05, r = .42).

#### 3.5.2 Load and neuropsychological performances

Load related connectivity was not related to executive processing in either group, but did predict episodic memory of the control group, where better CVLT scores were predicted by increased preSMA‐inferior frontal/insular cortex connectivity during retrieval (F = 15.04, *p* < .001, r = .61). In both groups, individual differences in visuospatial processing on the Benton Visual Form Test were predicted by load‐related connectivity. In the PD group, better visuospatial processing was predicted by stronger left middle frontal‐left occipital/fusiform connectivity during encoding (F = 5.20, q < .05, r = .42) and stronger preSMA—right inferior frontal/insula connectivity (F = 4.60, q < .05, r = .40) during retrieval. In the control group, better visuospatial processing was predicted by decreased right thalamus—left inferior/DLPFC connectivity during encoding (F = 17.38, *p* < .0004, r = −.66).

### 3.6 Sensitivity of aberrant context‐dependent measures

Discriminant analyses with classification were performed on two sets of PPI variables that showed group differences. Distraction‐related connectivity features (Table 2; 11 variables) correctly classified 93% of the controls and 90% of PD patients (Chi‐square = 58.2, *p* ≤ 1.99E−8). Load‐related connectivity features (Table 3; nine variables) correctly classified 90% of controls and 90% of PD patients (Chi‐square = 45.7, *p* ≤ 1.02E−9). The ROC analyses indicated that features of group differences showed excellent overall accuracy in distinguishing a PD patient from healthy individuals (Distraction: AUC ≥0.98; 95% confidence interval = 0.94–1.00; *p* ≤ 2.49E−10; Load: AUC ≥0.96; 95% confidence interval = 0.92–1.00; p ≤ 1.02E−9).

## 4 DISCUSSION

Despite normal WM performance in PD, we observed for the first time disturbances in distraction and load‐related connectivity that differed for encoding and retrieval. Two aberrant connectivity dynamics were observed, one related to a loss in normal context‐dependent connectivity and another characterized by upregulated connectivity of WM hubs for which the control group did not exhibit context‐specific connectivity. During encoding, a loss in striatal‐prefrontal coupling was found both when distraction was present and memory load was high. However, other long‐range connectivity disturbances were unique to resisting distraction or managing high memory loads, and differed for encoding and retrieval processes. During distraction connectivity was notably upregulated in PD for different WM hubs during encoding (middle/superior frontal, medial temporal, occipital) and retrieval (precuneus). As for high memory loads, connectivity of different hubs was markedly upregulated during encoding (middle frontal) and lost during retrieval (DLPFC, preSMA, and posterior cingulate). These findings demonstrate that aberrant neuromodulation in PD depends on the brain systems that are recruited by different cognitive processes. Abnormal functional connectivity was also behaviorally significant, with some connectivity features having pathological associations with WM performance and others suggesting possible compensation via amplified connections with visuospatial attention and object processing centers. Altered connectivity was not accompanied by group differences in distraction‐ or load‐related levels of regional activation, which largely aligns with a study of verbal WM load in cognitively normal PD (Poston et al., 2016). ROC analyses further demonstrated that group differences in connectivity distinguish PD from controls with high accuracy (AUC ≥ .96). Altogether, long‐range connectivity of WM hubs is profoundly altered in cognitively normal PD in a manner that depends on fundamental cognitive processes.

### 4.1 Abnormal distraction‐dependent connectivity

The influence of distraction on the connectivity of WM hubs in the control group was confined to the left putamen, wherein coupling was strengthened with the left superior frontal cortex. This result aligns with the basal ganglia's role in updating cognitive goals in concert with a region of the dorsal attention network (DAN) that supports top‐down visuospatial attention (Braga, Wilson, Sharp, Wise, & Leech, 2013; Corbetta, Patel, & Shulman, 2008). This frontostriatal circuit appears to set the system to selectively attend, thereby preventing undue interference from irrelevant stimuli (Ekman et al., 2016; Hazy et al., 2007; McNab & Klingberg, 2008). In contrast, PD participants failed to increase putamen—superior frontal cortex connectivity during distraction, despite advance cues signaling that an array would contain irrelevant shapes. This finding comports with the loss in intrinsic striatal connectivity in PD patients off, but not on dopaminergic therapy (Bell et al., 2015).

Rather, patients managed distractions during encoding by amplifying the long‐range connectivity of other prefrontal DAN hubs (BA 8, 6) that modulate visuospatial WM (Christophel et al., 2017; du Boisgueheneuc et al., 2006; Kastner et al., 2007). Right superior frontal cortex (BA 8) connectivity was abnormally strengthened with the basal‐ganglia, thalamic nuclei that support spatial WM (dorsomedial and pulvinar) (Funahashi, 2013; Nakajima & Halassa, 2017), an encoding and recollection system (precuneus, posterior cingulate, PHC) (Cavanna & Trimble, 2006; Ranganath & Ritchey, 2012), and the ventral object processing system (middle and superior temporal) (Vilberg & Rugg, 2012). Left middle frontal (BA 6) connectivity was abnormally strengthened with the right inferior parietal cortex, an association area that subserves spatial functions (Singh‐Curry & Husain, 2009). In addition, connectivity of a memory (right PHC) (Aminoff, Kveraga, & Bar, 2013) and object processing hub (left lingual gyrus) were amplified with the salience network (right insula) and left putamen, respectively. Notably, aberrant features of distraction‐related connectivity were predominantly characterized by upregulated cortico–cortical interactions, which support memory encoding and maintenance (Ekman et al., 2016). An exception was the finding of aberrantly strengthened lingual gyrus—putamen connectivity, which was associated with lower memory capacity in PD, possibly suggesting that too much reliance on perceptual processing regions renders the system more vulnerable to distraction. On the other hand, left middle frontal—right inferior parietal coupling was stronger in patients with better visuospatial skills, but not better WM. One speculation is that upregulated prefrontal connectivity with regions that deploy top‐down spatial attention may stabilize encoding and maintenance during distraction, thereby postponing memory decline (Reuter‐Lorenz & Park, 2014) without necessary correlating with individual differences in WM performance.

Upregulated connectivity of cortical WM hubs in PD in the face of distraction contrasted with controls who exhibited stronger cortical couplings during no distraction than distraction. Interestingly, stronger connectivity in the absence of distraction was observed for other WM hubs in both the control and PD groups. Notably, right middle frontal (BA 6)—left inferior parietal coupling was stronger during no distraction in both groups. Preserved connectivity within this circuit in the PD group contrasted with left middle frontal (BA 6)—right inferior parietal cortex connectivity, which was upregulated during distraction. Thus, opposite frontoparietal connectivity patterns were observed in homologous circuits that are pivotal for WM, which is compatible with compensation in PD when the demands on focused spatial attention are amplified (Ekman et al., 2016). Likewise, in both groups right putamen and bilateral precuneus couplings were stronger in the absence of distraction, possibly reflecting preserved updating (McNab & Klingberg, 2008) and storage (Galeano Weber et al., 2017) in PD when focusing attention is easier.

Entirely different features of aberrant connectivity in PD were uncovered when retrieving a target encoded in the face of distraction. Here, connectivity disturbances were specific to the bilateral precuneus, which together with other parietal‐occipital regions represent the precision by which items are remembered (Christophel et al., 2015; Galeano Weber et al., 2017; Schott et al., 2019; Wang, Itthipuripat, & Ku, 2019). Precuneus coupling was strengthened with prefrontal regions (BA 10) that control top‐down attention (Simons, Gilbert, Owen, Fletcher, & Burgess, 2005) and a memory recollection network (posterior cingulate, PHC) (Jonker, Dimsdale‐Zucker, Ritchey, Clarke, & Ranganath, 2018; Ranganath & Ritchey, 2012; Thakral, Wang, & Rugg, 2017). Moreover, stronger right precuneus—posterior cingulate connectivity was associated with poorer discriminability of targets, perhaps signifying diminished fidelity of visual representations that were encoded during distraction (Galeano Weber et al., 2017; Schott et al., 2019). In contrast, PD patients with better visual organization skills showed more marked coupling between the left precuneus and middle temporal cortex, which encodes object attributes (Vilberg & Rugg, 2012). Thus, a benefit of upregulating interactions within this circuit is that it may reactivate stored features of visual arrays, which could help sustain normal WM performance. Altogether, upregulated long‐range precuneus connectivity in PD may signify early memory retrieval difficulties.

### 4.2 Abnormal memory load‐dependent connectivity

As for load‐related connectivity during encoding, in both groups coupling of the right caudate with anterior cingulate was stronger for large arrays. Thus, communications between regions that have long been implicated in diverse facets of executive control (Grahn, Parkinson, & Owen, 2008; Shenhav & Mendes, 2014) were preserved in PD. However, other features of load‐related connectivity were abnormal in PD during the encoding phase, including interactions within different frontostriatal circuits. Patients failed to strengthen coupling of striatal‐thalamocortical hubs when encoding larger arrays, unlike the control group. This loss in connectivity resembled PD patients' loss in coupling of a similar circuit (left putamen—BA 8) when encoding during distraction. Both findings suggest that frontostriatal dysfunction disrupts flexible updating of WM (Ekman et al., 2016; Hazy et al., 2007; McNab & Klingberg, 2008). Although load‐related putamen coupling in the control group was found with more distributed prefrontal regions (BA 8, DLPFC), this finding may reflect the higher demands of updating four relevant shapes in comparison to arrays of the same size that contain two relevant items and two distractors (Badre & Nee, 2018; Linden et al., 2003; Nee & Brown, 2013). Interestingly, in the control group stronger load‐dependent coupling within this circuit was associated with poorer distraction resistance, signifying individual differences in the ability to accurately encode larger amounts of visuospatial information.

The PD group also failed to increase thalamus‐prefrontal connectivity (DLPFC, BA 10) when the burden on WM was greater, unlike the control group. The mediodorsal thalamus directs arousal in a context‐specific manner by amplifying local prefrontal cortex connectivity (Nakajima & Halassa, 2017; Schmitt et al., 2017), which serves to maintain representations active over time. PD patients who resembled control participants with respect to amplified load‐dependent mediodorsal thalamus connectivity also exhibited higher capacity and better distractor resistance. At the same time, patients managed larger memory loads by increasing connectivity of the left middle frontal cortex (BA 6) with precuneus, temporal, and occipital cortices. Stronger middle frontal—occipital/fusiform connectivity was also associated with better visual form discrimination, perhaps suggesting that amplified prefrontal connectivity with object‐identification systems (Ekman et al., 2016; Linden et al., 2003) might stabilize encoding and maintenance of visual representations.

As for retrieving a target that was stored in a large array, PD patients failed to amplify long‐range connectivity of key prefrontal WM hubs (DLPFC, preSMA). In the control group, strengthened DLPFC coupling with the precuneus comports with stronger DLPFC excitation of parietal cortex for higher memory loads (Edin et al., 2009). Yet, the PD group exhibited negative coupling within this circuit, which was recently found in a small cohort of de novo PD patients (*n* = 15) during a visual n‐back task (Trujillo et al., 2015). The central role that prefrontal–parietal networks normally play in memory retrieval was further underscored by the stronger load‐dependent connectivity of the preSMA with the precuneus in the control, but not PD group. In addition, upregulated preSMA connectivity with the salience network (inferior frontal and insular cortices) in the control, but not PD group was associated with better short‐term episodic memory, consistent with this circuit's role in memory retrieval. Interestingly, PD patients who resembled controls with respect to amplified preSMA‐salience network connectivity had better visual organization skills, potentially suggesting that this pathway may boost retrieval via reactivation of salient visual representations (Linden et al., 2003). Thus, another feature of retrieval difficulties in PD is the loss in long‐range connectivity of prefrontal cortex when visuospatial WM nears capacity limits. At the same time, load‐related connectivity in PD was preserved in different WM hubs (left BA 6, right precuneus, right putamen) when retrieving a target stored in a large array. In both groups, coupling of these WM hubs was stronger notably with visual (occipital) and memory retrieval (PC, precuneus) systems, which may help sustain memory retrieval in PD, at least for patients without clinically significant cognitive impairment.

### 4.3 Limitations

Our results may be partly related to testing patients off medication, which can produce greater disturbances in brain activation during verbal and visuospatial WM tasks (Poston et al., 2016; Simioni et al., 2017), although less so in cognitively normal PD cohorts (Poston et al., 2016). Indeed, despite the lingering effects of dopamine after short‐term medication withdrawal, levodopa dosage equivalence was not correlated with MRI or behavioral variables, suggesting that this factor may have not had a large effect on our findings. While drug naïve patients would be a more ideal group to study, we note that the loss in load‐related left DLPFC—precuneus coupling in our PD group replicated similar results in a study of de novo PD that specifically focused on context‐dependent connectivity of the left DLPFC during a visual n‐back task (Trujillo et al., 2015). Another limitation is that participants were more highly educated than is typical, so that the results may not generalize to all PD patients. While our recruitment methods were not biased toward more highly educated individuals, it is possible that greater cognitive reserve helps people to better cope with brain pathology by sustaining normal performance levels during cognitive testing (Hindle, Martyr, & Clare, 2014; Lucero et al., 2015). To fully address this issue, future studies should assess interactions of proxies for cognitive reserve on both behavior and brain function. An additional matter concerns the absence of group differences in the magnitude of distraction and load effects on working memory, despite robust effects of both manipulations on performance. These results are in accord with normal cognition in our PD cohort who was screened for MCI based on the Movement Disorders Society Level II criteria (Litvan et al., 2012). Nonetheless, other experimental designs might be more sensitive to behavioral decline in WM even in patients with normal cognition. For example, we speculated that the association between stronger precuneus‐posterior cingulate connectivity and worse discriminability when retrieving an item encoded during distraction might be due to reduced precision of visual memories. Hence, measures of recall precision (Galeano Weber et al., 2017) may be more sensitive to the quality of working memory, as suggested by a study of 12 PD on patients who were not screened for MCI (Zokaei, Burnett, Gorgoraptis, Budhdeo, & Husain, 2015). The ability to resist distraction can also be more difficult when it occurs immediately after than during encoding, as in our study (McNab et al., 2015). Still, distraction following encoding of faces and scenes failed to produce abnormal distraction costs in 15 PD off patients (Cools, Miyakawa, Sheridan, & D'Esposito, 2010), possibly because patients' ability to filter distractions critically depends on the complexity (i.e., level of structure) of visual configurations (Fallon, Bor, Hampshire, Barker, & Owen, 2017). These properties and others that amplify the difficulty of different visuospatial WM processes have been understudied in PD and deserve more consideration, especially in patients with normal cognition. A related issue is that other WM paradigms might also probe for context‐dependent connectivity in regions for which we did not observe distraction or load modulated connectivity. However, group differences in distraction and memory‐load related connectivity distinguished PD patients from controls with high accuracy (AUC ≥ .96), indicating that out experimental manipulations were robust probes for brain dysfunction. Lastly, neurocognitive correlations were typically medium in magnitude. While our study contains the largest cohort of cognitively normal PD patients studied to date during working‐memory evoked‐fMRI, larger sample sizes are desirable for testing neurocognitive relationships owing to the more restricted ranges on cognitive measures relative to studies of mixed patient samples, some with and others without MCI.

## 5 CONCLUSIONS

Despite robust effects of the experimental manipulations on regional activation, no group differences were found in the magnitude of distraction and memory load effects. Conventional regional analyses are limited because cognition depends upon interactions amongst brain regions that support behavior, rather than isolated processes within regions. A related issue is that neuropathological changes underlying WM in PD are also likely to be widely distributed, partly owing to altered dopamine and non‐dopamine neurotransmission (cholinergic, noradrenergic) (Gratwicke, Jahanshahi, & Foltynie, 2015). There is also considerable heterogeneity in cognitive functioning, including the domain(s) of cognition that decline in PD (Kehagia, Barker, & Robbins, 2010). These variables can render the connectivity of neural circuits or networks more vulnerable without necessarily affecting the intensity of regional activation, which may not be sufficiently or consistently altered across individuals (Rowe, 2010), especially cognitively normal patients. Thus, complementary analyses of functional connectivity should be used, as they may be important proxies of early cognitively‐related neuropathology. In this regard, multiple analytic approaches should be leveraged to build an understanding of brain functioning at different levels of analysis (e.g., gPPI, dynamic causal modeling, graph theory approaches, network‐based statistics). Results from one approach (e.g., gPPI) can also be used to inform other approaches (e.g., dynamic causal modeling, graph theory approaches). While abnormal connectivity features can be complex, studies aimed at validating outcomes are essential and may be refined by the application of data reduction methods (e.g., deep learning), which can render combinations of features more interpretable and diagnostic.

In this regard, the distinctive feature of WM‐related connectivity in PD was abnormal frontal‐posterior cortical and corticostriatal coupling, despite normal WM performance. Patterns of abnormal coupling and uncoupling of WM hubs in PD differed in the face of distraction and high memory loads, and were also distinct for encoding and retrieval. Difficulties in suppressing distraction were suggested by upregulated connectivity of WM hubs during distraction, yet the same hubs exhibited decreased connectivity during distraction in controls. In contrast, difficulties in managing larger memory loads were revealed by abnormally upregulated and down regulated connectivity of WM hubs during encoding and retrieval, respectively. These results underscored the importance characterizing brain dysfunction as it relates to specific inherent WM processes. This in turn should improve the sensitivity of studies aimed at tracking changes in different networks and better inform treatments that attack neurodegeneration at its earliest stage.

We also found that some aberrant connectivity dynamics were pathological as they were associated with poorer WM. Aberrant connectivity for other hubs was not associated with WM proficiency, perhaps suggesting that functional reorganization does not always support cognition. However, other mechanisms may also explain these findings. One possibility is that upregulated connectivity signifies difficulties in modulating interactions of WM hubs during effortful cognition, owing to reduced fidelity or coherence within functionally reorganized systems, which should increase trial‐by‐trial variability such that connectivity would not necessarily correlate with performance. Alternatively, aberrantly strengthened connectivity could be compensatory. For example, upregulated connectivity may improve long‐range communications of WM hubs, thereby potentially masking or postponing the onset of WM decline (Reuter‐Lorenz & Park, 2014). In this regard, we also found that abnormally strengthened connectivity of different hubs was associated with better visuospatial processing in PD, which may compensate for subtle WM difficulties by amplifying connectivity with spatial attention and object processing regions that could help stabilize or reactivate encoded representations. These sorts of compensatory mechanisms would not necessarily produce correlations with WM proficiency, although this prospect needs to be examined using a longitudinal study design. Still, larger PD cohorts are needed to better evaluate the behavioral significance of aberrant connectivity features. Altogether, our results offer new insights into the neural signatures of aberrant context‐specific WM processes in PD, which may presage future declines in visuospatial WM. Since treatment interventions are more likely to be effective before neuropathology progresses, studies are needed that rigorously screen for PD MCI to build an understanding of early changes in WM networks that support specific processes, independent of more generalized cognitive decline. This knowledge is more likely to inform targeted therapeutic interventions and may aid in identifying appropriate patients for clinical trials.

## CONFLICT OF INTEREST

The authors do not have any conflicts of interest to declare.

## Supporting information


**Table S1** Significant effects of distraction on regional activation^1^ and group differences in regional activation.^2^
**Table S2.** Significant effects of memory load on regional activation^1^ and group differences in regional activation.^2^
**Table S3.** Seed coordinates based on peak activation in clusters that were significantly associated with distraction and memory load effects in voxelwise analyses of all subjects.
**Figure S1.** Regions of interest showing no group differences for distraction or memory load manipulations. The figure illustrates two different patterns of connectivity results that did not differ between the PD and control groups. Purple circles show seed ROI that did not exhibit distraction‐related (top row) or memory load‐related (bottom row) connectivity in either group (i.e., failed to reach the thresholded of a voxelwise‐probability of *p* < 0.005 and minimum cluster size of 41 voxels). Blue circles display seed ROI that showed significant context‐dependent with other brain regions (gold circles and lines), which did not differ between the PD and control groups. The top row shows that in both groups distraction dependent connectivity was greater during no distraction than distraction during the encoding phase. The bottom row shows that in both groups load‐dependent connectivity was greater for 4 than 2 shape arrays during the encoding and retrieval phases. Tables 2 and 3 provide the coordinates for distraction and load‐related connectivity features that did not differ between groups.
DLPFC = dorsal lateral prefrontal cortex; IPL = inferior parietal lobule; MFG = middle frontal gyrus (BA 6); MO = middle occipital (BA 19); Par = paracentral cortex; PC = posterior cingulate; Precun = precuneus; pPHC = posterior parahippocampal cortex; pPHC = posterior parahippocampal cortex; Put = putamen; SMA = supplementary motor area; Thal = thalamus

## Data Availability

Data that support the findings of this study are available from the corresponding author upon reasonable request. The data are not publically available due to privacy or ethical restrictions.

## References

[hbm24868-bib-0001] Aminoff, E. M. , Kveraga, K. , & Bar, M. (2013). The role of the parahippocampal cortex in cognition. Trends in Cognitive Sciences, 17, 379–390. 10.1016/j.tics.2013.06.009 23850264 PMC3786097

[hbm24868-bib-0002] Badre, D. , & Nee, D. E. (2018). Frontal cortex and the hierarchical control of behavior. Trends in Cognitive Sciences, 22, 170–188. 10.1016/j.tics.2017.11.005 29229206 PMC5841250

[hbm24868-bib-0003] Beall, E. B. , & Lowe, M. J. (2014). SimPACE: Generating simulated motion corrupted BOLD data with synthetic‐navigated acquisition for the development and evaluation of SLOMOCO: A new, highly effective slicewise motion correction. NeuroImage, 101, 21–34. 10.1016/j.neuroimage.2014.06.038 24969568 PMC4165749

[hbm24868-bib-0004] Bell, P. T. , Gilat, M. , O'Callaghan, C. , Copland, D. A. , Frank, M. J. , Lewis, S. J. , & Shine, J. M. (2015). Dopaminergic basis for impairments in functional connectivity across subdivisions of the striatum in Parkinson's disease. Human Brain Mapping, 36, 1278–1291. 10.1002/hbm.22701 25425542 PMC6869546

[hbm24868-bib-0005] Braga, R. M. , Wilson, L. R. , Sharp, D. J. , Wise, R. J. , & Leech, R. (2013). Separable networks for top‐down attention to auditory non‐spatial and visuospatial modalities. NeuroImage, 74, 77–86. 10.1016/j.neuroimage.2013.02.023 23435206 PMC3898942

[hbm24868-bib-0006] Caminiti, S. P. , Siri, C. , Guidi, L. , Antonini, A. , & Perani, D. (2015). The neural correlates of spatial and object working memory in elderly and Parkinson's disease subjects. Behavioural Neurology, 2015, 1–10. 10.1155/2015/123636 PMC4378329 25861157

[hbm24868-bib-0007] Cavanna, A. E. , & Trimble, M. R. (2006). The precuneus: A review of its functional anatomy and behavioural correlates. Brain, 129, 564–583. 10.1093/brain/awl004 16399806

[hbm24868-bib-0008] Chen, G. , Adleman, N. E. , Saad, Z. S. , Leibenluft, E. , & Cox, R. W. (2014). Applications of multivariate modeling to neuroimaging group analysis: A comprehensive alternative to univariate general linear model. NeuroImage, 99, 571–588. 10.1016/j.neuroimage.2014.06.027 24954281 PMC4121851

[hbm24868-bib-0009] Christophel, T. B. , Cichy, R. M. , Hebart, M. N. , & Haynes, J. D. (2015). Parietal and early visual cortices encode working memory content across mental transformations. NeuroImage, 106, 198–206. 10.1016/j.neuroimage.2014.11.018 25463456

[hbm24868-bib-0010] Christophel, T. B. , Klink, P. C. , Spitzer, B. , Roelfsema, P. R. , & Haynes, J. D. (2017). The distributed nature of working memory. Trends in Cognitive Sciences, 21, 111–124. 10.1016/j.tics.2016.12.007 28063661

[hbm24868-bib-0011] Cisler, J. M. , Bush, K. , & Steele, J. S. (2014). A comparison of statistical methods for detecting context‐modulated functional connectivity in fMRI. NeuroImage, 84, 1042–1052. 10.1016/j.neuroimage.2013.09.018 24055504 PMC4019671

[hbm24868-bib-0012] Cools, R. , & D'Esposito, M. (2011). Inverted‐U‐shaped dopamine actions on human working memory and cognitive control. Biological Psychiatry, 69, e113–e125. 10.1016/j.biopsych.2011.03.028 21531388 PMC3111448

[hbm24868-bib-0013] Cools, R. , Miyakawa, A. , Sheridan, M. , & D'Esposito, M. (2010). Enhanced frontal function in Parkinson's disease. Brain, 133, 225–233. 10.1093/brain/awp301 19995871 PMC2801327

[hbm24868-bib-0014] Corbetta, M. , Patel, G. , & Shulman, G. L. (2008). The reorienting system of the human brain: From environment to theory of mind. Neuron, 58, 306–324. 10.1016/j.neuron.2008.04.017 18466742 PMC2441869

[hbm24868-bib-0015] Cox, R. W. , Chen, G. , Glen, D. R. , Reynolds, R. C. , & Taylor, P. A. (2017a). fMRI clustering and false‐positive rates. Proceedings of the National Academy of Sciences of the United States of America, 114, E3370–E3371. 10.1073/pnas.1614961114 28420798 PMC5410825

[hbm24868-bib-0016] Cox, R. W. , Chen, G. , Glen, D. R. , Reynolds, R. C. , & Taylor, P. A. (2017b). FMRI clustering in AFNI: False‐positive rates redux. Brain Connectivity, 7, 152–171. 10.1089/brain.2016.0475 28398812 PMC5399747

[hbm24868-bib-0017] du Boisgueheneuc, F. , Levy, R. , Volle, E. , Seassau, M. , Duffau, H. , Kinkingnehun, S. , … Dubois, B. (2006). Functions of the left superior frontal gyrus in humans: A lesion study. Brain, 129, 3315–3328. 10.1093/brain/awl244 16984899

[hbm24868-bib-0018] Edin, F. , Klingberg, T. , Johansson, P. , McNab, F. , Tegner, J. , & Compte, A. (2009). Mechanism for top‐down control of working memory capacity. Proceedings of the National Academy of Sciences of the United States of America, 106, 6802–6807. 10.1073/pnas.0901894106 19339493 PMC2672558

[hbm24868-bib-0019] Ekman, M. , Fiebach, C. J. , Melzer, C. , Tittgemeyer, M. , & Derrfuss, J. (2016). Different roles of direct and indirect Frontoparietal pathways for individual working memory capacity. The Journal of Neuroscience, 36, 2894–2903. 10.1523/JNEUROSCI.1376-14.2016 26961945 PMC6601754

[hbm24868-bib-0020] Ekman, U. , Eriksson, J. , Forsgren, L. , Mo, S. J. , Riklund, K. , & Nyberg, L. (2012). Functional brain activity and presynaptic dopamine uptake in patients with Parkinson's disease and mild cognitive impairment: A cross‐sectional study. Lancet Neurol, 11, 679–687. 10.1016/S1474-4422(12)70138-2 22742929

[hbm24868-bib-0021] Fallon, S. J. , Bor, D. , Hampshire, A. , Barker, R. A. , & Owen, A. M. (2017). Spatial structure normalises working memory performance in Parkinson's disease. Cortex, 96, 73–82. 10.1016/j.cortex.2017.08.023 28985531

[hbm24868-bib-0022] Fallon, S. J. , Mattiesing, R. M. , Muhammed, K. , Manohar, S. , & Husain, M. (2017). Fractionating the neurocognitive mechanisms underlying working memory: Independent effects of dopamine and Parkinson's disease. Cerebral Cortex, 27, 5727–5738. 10.1093/cercor/bhx242 29040416 PMC5939219

[hbm24868-bib-0023] Funahashi, S. (2013). Thalamic mediodorsal nucleus and its participation in spatial working memory processes: Comparison with the prefrontal cortex. Frontiers in Systems Neuroscience, 7:36. https://doi.org/fnsys.2013.00036 23914160 10.3389/fnsys.2013.00036 PMC3728470

[hbm24868-bib-0024] Galeano Weber, E. M. , Hahn, T. , Hilger, K. , & Fiebach, C. J. (2017). Distributed patterns of occipito‐parietal functional connectivity predict the precision of visual working memory. NeuroImage, 146, 404–418. 10.1016/j.neuroimage.2016.10.006 27721028

[hbm24868-bib-0025] Galeano Weber, E. M. , Peters, B. , Hahn, T. , Bledowski, C. , & Fiebach, C. J. (2016). Superior intraparietal sulcus controls the variability of visual working memory precision. The Journal of Neuroscience, 36, 5623–5635. 10.1523/JNEUROSCI.1596-15.2016 27194340 PMC6601772

[hbm24868-bib-0026] Grahn, J. A. , Parkinson, J. A. , & Owen, A. M. (2008). The cognitive functions of the caudate nucleus. Progress in Neurobiology, 86, 141–155. 10.1016/j.pneurobio.2008.09.004 18824075

[hbm24868-bib-0027] Gratwicke, J. , Jahanshahi, M. , & Foltynie, T. (2015). Parkinson's disease dementia: A neural networks perspective. Brain, 138, 1454–1476. 10.1093/brain/awv104 25888551 PMC4614131

[hbm24868-bib-0028] Hazy, T. E. , Frank, M. J. , & O'Reilly, R. C. (2007). Towards an executive without a homunculus: Computational models of the prefrontal cortex/basal ganglia system. Philosophical Transactions of the Royal Society of London. Series B, Biological Sciences, 362, 1601–1613. 10.1098/rstb.2007.2055 17428778 PMC2440774

[hbm24868-bib-0070] Hindle, J.V. , Martyr, A. , Clare, L. (2014). Cognitive reserve in Parkinson's disease: a systematic review and meta‐analysis. Parkinsonism Relat Disord, 20, 1–7. https://doi.org/S1353-8020(13)00304-0. 24034887 10.1016/j.parkreldis.2013.08.010

[hbm24868-bib-0029] Ichihara‐Takeda, S. , & Funahashi, S. (2007). Activity of primate orbitofrontal and dorsolateral prefrontal neurons: Task‐related activity during an oculomotor delayed‐response task. Experimental Brain Research, 181, 409–425. 10.1007/s00221-007-0941-0 17443317

[hbm24868-bib-0030] Johnson, E. L. , Dewar, C. D. , Solbakk, A. K. , Endestad, T. , Meling, T. R. , & Knight, R. T. (2017). Bidirectional Frontoparietal oscillatory systems support working memory. Current Biology, 27, 1829–1835. 10.1016/j.cub.2017.05.046 28602658 PMC5546232

[hbm24868-bib-0031] Jonker, T. R. , Dimsdale‐Zucker, H. , Ritchey, M. , Clarke, A. , & Ranganath, C. (2018). Neural reactivation in parietal cortex enhances memory for episodically linked information. Proceedings of the National Academy of Sciences of the United States of America, 115, 11084–11089. 10.1073/pnas.1800006115 PMC6205442 30297400

[hbm24868-bib-0032] Kastner, S. , DeSimone, K. , Konen, C. S. , Szczepanski, S. M. , Weiner, K. S. , & Schneider, K. A. (2007). Topographic maps in human frontal cortex revealed in memory‐guided saccade and spatial working‐memory tasks. Journal of Neurophysiology, 97, 3494–3507. 10.1152/jn.00010.2007 17360822

[hbm24868-bib-0033] Kehagia, A. A. , Barker, R. A. , & Robbins, T. W. (2010). Neuropsychological and clinical heterogeneity of cognitive impairment and dementia in patients with Parkinson's disease. Lancet Neurology, 9, 1200–1213. 10.1016/S1474-4422(10)70212-X 20880750

[hbm24868-bib-0034] Lee, E. Y. , Cowan, N. , Vogel, E. K. , Rolan, T. , & Valle‐Inc, H. S. A. (2010). Visual working memory deficits in patients with Parkinson's disease are due to both reduced storage capacity and impaired ability to filter out irrelevant information. Brain, 133, 2677–2689. 10.1093/brain/awq197 20688815 PMC2929336

[hbm24868-bib-0035] Lewis, S. J. , Dove, A. , Robbins, T. W. , Barker, R. A. , & Owen, A. M. (2003). Cognitive impairments in early Parkinson's disease are accompanied by reductions in activity in frontostriatal neural circuitry. The Journal of Neuroscience, 23, 6351–6356. 10.1523/JNEUROSCI.23-15-06351.2003 12867520 PMC6740550

[hbm24868-bib-0036] Linden, D. E. , Bittner, R. A. , Muckli, L. , Waltz, J. A. , Kriegeskorte, N. , Goebel, R. , … Munk, M. H. (2003). Cortical capacity constraints for visual working memory: Dissociation of fMRI load effects in a fronto‐parietal network. NeuroImage, 20, 1518–1530. 10.1016/j.neuroimage.2003.07.021 14642464

[hbm24868-bib-0037] Litvan, I. , Goldman, J. G. , Troster, A. I. , Schmand, B. A. , Weintraub, D. , Petersen, R. C. , … Emre, M. (2012). Diagnostic criteria for mild cognitive impairment in Parkinson's disease: Movement Disorder Society task force guidelines. Movement Disorders, 27, 349–356. 10.1002/mds.24893 22275317 PMC3641655

[hbm24868-bib-0071] Lucero, C. , Campbell, M.C. , Flores, H. , Maiti, B. , Perlmutter, J.S. , Foster, E.R. (2015). Cognitive reserve and beta‐amyloid pathology in Parkinson disease. Parkinsonism Relat Disord, 21, 899–904. 10.1016/j.parkreldis.2015.05.020. 26037458 PMC4509801

[hbm24868-bib-0038] Marklund, P. , Larsson, A. , Elgh, E. , Linder, J. , Riklund, K. A. , Forsgren, L. , & Nyberg, L. (2009). Temporal dynamics of basal ganglia under‐recruitment in Parkinson's disease: Transient caudate abnormalities during updating of working memory. Brain, 132, 336–346. 10.1093/brain/awn309 19036762

[hbm24868-bib-0039] Mattay, V. S. , Tessitore, A. , Callicott, J. H. , Bertolino, A. , Goldberg, T. E. , Chase, T. N. , … Weinberger, D. R. (2002). Dopaminergic modulation of cortical function in patients with Parkinson's disease. Annals of Neurology, 51, 156–164. 10.1002/ana.10078 11835371

[hbm24868-bib-0040] McLaren, D. G. , Ries, M. L. , Xu, G. , & Johnson, S. C. (2012). A generalized form of context‐dependent psychophysiological interactions (gPPI): A comparison to standard approaches. NeuroImage, 61, 1277–1286. 10.1016/j.neuroimage.2012.03.068 22484411 PMC3376181

[hbm24868-bib-0041] McNab, F. , & Klingberg, T. (2008). Prefrontal cortex and basal ganglia control access to working memory. Nature Neuroscience, 11, 103–107. 10.1038/nn2024 18066057

[hbm24868-bib-0042] McNab, F. , Leroux, G. , Strand, F. , Thorell, L. , Bergman, S. , & Klingberg, T. (2008). Common and unique components of inhibition and working memory: An fMRI, within‐subjects investigation. Neuropsychologia, 46, 2668–2682. 10.1016/j.neuropsychologia.2008.04.023 18573510

[hbm24868-bib-0043] McNab, F. , Zeidman, P. , Rutledge, R. B. , Smittenaar, P. , Brown, H. R. , Adams, R. A. , & Dolan, R. J. (2015). Age‐related changes in working memory and the ability to ignore distraction. Proc Natl Acad Sci U S A, 112, 6515–6518. 10.1073/pnas.1504162112 25941369 PMC4443336

[hbm24868-bib-0044] Murray, J. D. , Jaramillo, J. , & Wang, X. J. (2017). Working memory and decision‐making in a Frontoparietal circuit model. The Journal of Neuroscience, 37, 12167–12186. 10.1523/JNEUROSCI.0343-17.2017 PMC5729190 29114071

[hbm24868-bib-0045] Murty, V. P. , Sambataro, F. , Radulescu, E. , Altamura, M. , Iudicello, J. , Zoltick, B. , … Mattay, V. S. (2011). Selective updating of working memory content modulates meso‐cortico‐striatal activity. NeuroImage, 57, 1264–1272. 10.1016/j.neuroimage.2011.05.006 21596142 PMC3908780

[hbm24868-bib-0046] Muslimovic, D. , Post, B. , Speelman, J. D. , & Schmand, B. (2005). Cognitive profile of patients with newly diagnosed Parkinson disease. Neurology, 65, 1239–1245. 10.1212/01.wnl.0000180516.69442.95 16247051

[hbm24868-bib-0047] Nakajima, M. , & Halassa, M. M. (2017). Thalamic control of functional cortical connectivity. Current Opinion in Neurobiology, 44, 127–131. 10.1016/j.conb.2017.04.001 28486176 PMC5604244

[hbm24868-bib-0048] Nee, D. E. , & Brown, J. W. (2013). Dissociable frontal‐striatal and frontal‐parietal networks involved in updating hierarchical contexts in working memory. Cerebral Cortex, 23, 2146–2158. 10.1093/cercor/bhs194 22798339 PMC3841420

[hbm24868-bib-0049] Pillon, B. , Deweer, B. , Agid, Y. , & Dubois, B. (1993). Explicit memory in Alzheimer's, Huntington's, and Parkinson's diseases. Archives of Neurology, 50, 374–379. 10.1001/archneur.1993.000540040036010 8460958

[hbm24868-bib-0050] Poston, K. L. , YorkWilliams, S. , Zhang, K. , Cai, W. , Everling, D. , Tayim, F. M. , … Menon, V. (2016). Compensatory neural mechanisms in cognitively unimpaired Parkinson disease. Annals of Neurology, 79, 448–463. 10.1001/10.1002/ana.24585 26696272 PMC4789131

[hbm24868-bib-0051] Ranchet, M. , Paire‐Ficout, L. , Marin‐Lamellet, C. , Laurent, B. , & Broussolle, E. (2011). Impaired updating ability in drivers with Parkinson's disease. Journal of Neurology, Neurosurgery, and Psychiatry, 82, 218–223. 10.1001/10.1136/jnnp.2009.203166 20802214

[hbm24868-bib-0052] Ranganath, C. , & Ritchey, M. (2012). Two cortical systems for memory‐guided behaviour. Nature Reviews. Neuroscience, 13, 713–726. 10.1001/10.1038/nrn3338 22992647

[hbm24868-bib-0053] Reuter‐Lorenz, P. A. , & Park, D. C. (2014). How does it STAC up? Revisiting the scaffolding theory of aging and cognition. Neuropsychology Review, 24, 355–370. 10.1007/s11065-014-9270-9 25143069 PMC4150993

[hbm24868-bib-0054] Rowe, J. B. (2010). Connectivity analysis is essential to understand neurological disorders. Frontiers in Systems Neuroscience, 4:144 10.1001/10.3389/fnsys.2010.00144 20948582 PMC2953412

[hbm24868-bib-0055] Schmitt, L. I. , Wimmer, R. D. , Nakajima, M. , Happ, M. , Mofakham, S. , & Halassa, M. M. (2017). Thalamic amplification of cortical connectivity sustains attentional control. Nature, 545, 219–223. 10.1001/10.1038/nature22073 28467827 PMC5570520

[hbm24868-bib-0056] Schott, B. H. , Wustenberg, T. , Lucke, E. , Pohl, I. M. , Richter, A. , Seidenbecher, C. I. , … Richardson‐Klavehn, A. (2019). Gradual acquisition of visuospatial associative memory representations via the dorsal precuneus. Human Brain Mapping, 40, 1554–1570. 10.1001/10.1002/hbm.24467 30430687 PMC6865719

[hbm24868-bib-0057] Shenhav, A. , & Mendes, W. B. (2014). Aiming for the stomach and hitting the heart: Dissociable triggers and sources for disgust reactions. Emotion, 14, 301–309. 10.1001/10.1037/a0034644 24219399 PMC4050063

[hbm24868-bib-0058] Siegert, R. J. , Weatherall, M. , Taylor, K. D. , & Abernethy, D. A. (2008). A meta‐analysis of performance on simple span and more complex working memory tasks in Parkinson's disease. Neuropsychol, 22, 450–461. 10.1001/10.1037/0894-4105.22.4.450 18590357

[hbm24868-bib-0059] Simioni, A. C. , Dagher, A. , & Fellows, L. K. (2017). Effects of levodopa on corticostriatal circuits supporting working memory in Parkinson's disease. Cortex, 93, 193–205. 10.1016/j.cortex.2017.05.021 28675834

[hbm24868-bib-0060] Simons, J. S. , Gilbert, S. J. , Owen, A. M. , Fletcher, P. C. , & Burgess, P. W. (2005). Distinct roles for lateral and medial anterior prefrontal cortex in contextual recollection. Journal of Neurophysiology, 94, 813–820. 10.1152/jn.01200.2004 15728761 PMC3838933

[hbm24868-bib-0061] Singh‐Curry, V. , & Husain, M. (2009). The functional role of the inferior parietal lobe in the dorsal and ventral stream dichotomy. Neuropsychologia, 47, 1434–1448. 10.1016/j.neuropsychologia.2008.11.033 19138694 PMC2697316

[hbm24868-bib-0062] Stolwyk, R. J. , Charlton, J. L. , Triggs, T. J. , Iansek, R. , & Bradshaw, J. L. (2006). Neuropsychological function and driving ability in people with Parkinson's disease. Journal of Clinical and Experimental Neuropsychology, 28, 898–913. 10.1080/13803390591000909 16822731

[hbm24868-bib-0063] Thakral, P. P. , Wang, T. H. , & Rugg, M. D. (2017). Decoding the content of recollection within the core recollection network and beyond. Cortex, 91, 101–113. 10.1016/j.cortex.2016.12.011 28077212 PMC5446805

[hbm24868-bib-0072] Tomlinson, C.L. , Stowe, R. , Patel, S. , Rick, C. , Gray, R. , Clarke, C.E. (2010). Systematic review of levodopa dose equivalency reporting in Parkinson's disease. Mov Disord, 25, 2649–2653. 10.1002/mds.23429. 21069833

[hbm24868-bib-0064] Trujillo, J. P. , Gerrits, N. J. , Veltman, D. J. , Berendse, H. W. , van der Werf, Y. D. , & van den Heuvel, O. A. (2015). Reduced neural connectivity but increased task‐related activity during working memory in de novo Parkinson patients. Human Brain Mapping, 36, 1554–1566. 10.1002/hbm.22723 25598397 PMC6869711

[hbm24868-bib-0065] Uc, E. Y. , Rizzo, M. , Anderson, S. W. , Sparks, J. D. , Rodnitzky, R. L. , & Dawson, J. D. (2006). Driving with distraction in Parkinson disease. Neurology, 67, 1774–1780. 10.1212/01.wnl.0000245086.32787.61 17130409

[hbm24868-bib-0066] Vilberg, K. L. , & Rugg, M. D. (2012). The neural correlates of recollection: Transient versus sustained FMRI effects. The Journal of Neuroscience, 32, 15679–15687. 10.1523/JNEUROSCI.3065-12.2012 PMC3509997 23136408

[hbm24868-bib-0067] Vogel, E. K. , McCollough, A. W. , & Machizawa, M. G. (2005). Neural measures reveal individual differences in controlling access to working memory. Nature, 438, 500–503. 10.1038/nature04171 16306992

[hbm24868-bib-0068] Wang, S. , Itthipuripat, S. , & Ku, Y. (2019). Electrical stimulation over human posterior parietal cortex selectively enhances the capacity of visual short‐term memory. The Journal of Neuroscience, 39, 528–536. 10.1523/JNEUROSCI.1959-18.2018 30459222 PMC6335754

[hbm24868-bib-0069] Zokaei, N. , Burnett, H. S. , Gorgoraptis, N. , Budhdeo, S. , & Husain, M. (2015). Working memory recall precision is a more sensitive index than span. Journal of Neuropsychology, 9, 319–329. 25208525 10.1111/jnp.12052

