# Supplementary material for: Abnormal distraction and load‐specific connectivity during working memory in cognitively normal Parkinson's disease
Source: Hum Brain Mapp. 2019 Nov 18;41(5):1195–211. doi: 10.1002/hbm.24868 (PMC7058508; doi:10.1002/hbm.24868)
Supplement: Supplementary file 1 — Table S1 Significant effects of distraction on regional activation1 and group differences in regional activation.2 Table S2. Significant effects of memory load on regional activation1 and group differences in regional activation.2 Table S3. Seed coordinates based on peak activation in clusters that were significantly associated with distraction and memory load effects in voxelwise analyses of all subjects. Figure S1. Regions of interest showing no group differences for distraction or memory load manipulations. The figure illustrates two different patterns of connectivity results that did not differ between the PD and control groups. Purple circles show seed ROI that did not exhibit distraction‐related (top row) or memory load‐related (bottom row) connectivity in either group (i.e., failed to reach the thresholded of a voxelwise‐probability of p < 0.005 and minimum cluster size of 41 voxels). Blue circles display seed ROI that showed significant context‐dependent with other brain regions (gold circles and lines), which did not differ between the PD and control groups. The top row shows that in both groups distraction dependent connectivity was greater during no distraction than distraction during the encoding phase. The bottom row shows that in both groups load‐dependent connectivity was greater for 4 than 2 shape arrays during the encoding and retrieval phases. Tables 2 and 3 provide the coordinates for distraction and load‐related connectivity features that did not differ between groups. DLPFC = dorsal lateral prefrontal cortex; IPL = inferior parietal lobule; MFG = middle frontal gyrus (BA 6); MO = middle occipital (BA 19); Par = paracentral cortex; PC = posterior cingulate; Precun = precuneus; pPHC = posterior parahippocampal cortex; pPHC = posterior parahippocampal cortex; Put = putamen; SMA = supplementary motor area; Thal = thalamus [file HBM-41-1195-s001.docx]

**SUPPLEMENTARY MATERIALS**

Table S1. Significant effects of distraction on regional activation^1^ and group differences in regional activation.^2^

| **Encoding Phase** | | | | |
| --- | --- | --- | --- | --- |
| **Distraction > No Distraction** | **X, Y, Z** | **Voxels** | **p value** | **η_p_^2^** |
| B precuneus, cuneus, lingual gyrus, cerebellum | -6 -78 -4 | 2386 | **0.04** | 0.07 |
| R MFG (BA 6) | 28 1 55 | 219 | 0.12 | 0.04 |
| L MFG (BA 6, 9) | -24 1 60 | 178 | 0.24 | 0.02 |
| R pPHC | 23 -61 -5 | 32 | 0.29 | 0.02 |
| L pPHC | -24 -61 -5 | 26 | 0.14 | 0.04 |
| R putamen, GP | 23 11 4 | 59 | 0.02 | 0.09 |
| L putamen, GP | -19 11 4 | 30 | 0.37 | 0.01 |
| **No Distraction > Distraction** |  |  |  |  |
| B SFG (BA 8,9) | 2 45 43 | 154 | 0.86 | 0.00 |
| L aPHC | -19 -19 -21 | 31 | 0.07 | 0.06 |
| **Retrieval Phase** | | | | |
| **Distraction > No Distraction** |  |  |  |  |
| B precuneus, SPL | 2 -60 73 | 181 | **0.001*** | 0.17 |
| R precuneus, SPL | 28 -70 46 | 55 | **0.01*** | 0.11 |
| **No Distraction > Distraction** |  |  |  |  |
| B SMA, L SM | -41 -19 52 | 301 | **0.015*** | 0.10 |
| R lingual gyrus | 11 -78 -8 | 39 | 0.19 | 0.03 |
| L putamen, GP | -24 10 -5 | 44 | 0.72 | 0.00 |
| R putamen, GP | 28 10 -1 | 36 | 0.11 | 0.05 |

^1^ Montreal Neurological Institute (MNI) atlas coordinates for regions showing significant effects of distraction in voxelwise analyses that were conducted for all participants combined. Thresholds for significance used a voxelwise probability of p < 0.0001 and a minimum cluster size of ≥ 38 voxels for cortex and p < 0.01 and a minimum cluster size of > 20 voxels for the striatum and parahippocampus.

^2^ ANCOVAs tested for group differences in regions that showed significant distraction effects. Group by distraction interactions were not significant for any regions. P values and η_p_^2^ values respectively designate significant main effects of group (p < 0.05, uncorrected) and associated effect sizes. Bolded p values with an asterisk are regions for which activation was significantly greater in the control than the PD group, regardless of distraction, after FDR adjustment for multiple comparisons.

B = bilateral hemispheres; L and R = left and right hemispheres; BA = Brodmann area; BG = basal ganglia; GP = globus pallidus; MFG = middle frontal gyrus; a/pPHC = anterior/posterior parahippocampus cortex; post = posterior; SFG = superior frontal gyrus; SM = sensorimotor; SMA = supplementary motor area; SPL = superior parietal lobule

Table S2. Significant effects of memory load on regional activation^1^ and group differences in regional activation.^2^

| **Encoding Phase** | | | | | | |
| --- | --- | --- | --- | --- | --- | --- |
| **4 > 2 array size** | **X, Y, Z^1^** | **Voxels** | | | **p value^2^** | **η_p_^2^** |
| B precuneus, SPL, occipital | 11 -90 6 | 2167 | | | 0.04 | 0.07 |
| B MFG (BA 6), B SMA, B putamen,  R caudate, R mdThal | 23 11 4 | 1243 | | | 0.02 | 0.08 |
| R DLPFC (BA 9) | 40 44 24 | 45 | | | 0.04 | 0.07 |
| R pPHC | 23 -61 -5 | 49 | | | 0.40 | 0.01 |
| L pPHC | -24 -61 -5 | 37 | | | 0.32 | 0.02 |
| **2 > 4 array size** |  |  | | |  |  |
| R SFG, mFG (BA 8) | 6 45 43 | 68 | | | 0.91 | 0.00 |
| **Retrieval Phase** | | | | | | |
| **4 > 2 array size** |  | |  |  | |  |
| B precuneus, IPL | 32 -66 41 | | 639 | 0.006 | | 0.13 |
| L MFG (BA 9,6), IFG | -41 10 54 | | 342 | 0.08 | | 0.05 |
| B preSMA (BA 6) | -6 22 49 | | 179 | 0.33 | | 0.02 |
| R DLPFC (BA 9) | 49 33 30 | | 78 | 0.01 | | 0.10 |
| R MFG (BA 6) | 32 10 54 | | 76 | 0.13 | | 0.04 |
| R IFG, insula | 36 26 -7 | | 58 | 0.08 | | 0.05 |
| **2 > 4 array size** |  | |  |  | |  |
| B cingulate, SMA, pre/post-central gyrus | -41 -23 52 | | 771 | 0.15 | | 0.04 |
| R IPL | 57 -29 30 | | 58 | 0.41 | | 0.01 |
| B post cingulate | -6 -46 26 | | 38 | 0.81 | | 0.00 |
| R putamen, GP | 23 10 -1 | | 71 | 0.55 | | 0.01 |
| L putamen, GP | -24 10 -1 | | 51 | 0.80 | | 0.00 |

^1^ Montreal Neurological Institute (MNI) atlas coordinates for regions showing significant effects of memory load in voxelwise analyses that were conducted for all participants combined. Thresholds for significance used a voxelwise probability of p < 0.0001 and a minimum cluster size of ≥ 38 voxels for cortex and p < 0.01 and a minimum cluster size of > 20 voxels for the striatum and parahippocampus.

^2^ ANCOVAs tested for group differences in regions that showed significant memory load effects. Group by memory load interactions were not significant for any regions. P values and η_p_^2^ values respectively designate significant main effects of group (p < 0.05, uncorrected) and associated effect sizes. No group differences were found after FDR adjustment for multiple comparisons.

B = bilateral hemispheres; L and R = left and right hemispheres; BA = Brodmann area; DLPFC = dorsal lateral prefrontal cortex; GP = globus pallidus; IFG = inferior frontal gyrus; IPL = inferior parietal lobule; mdThal = mediodorsal thalamus; mFG = medial frontal gyrus; MFG = middle frontal gyrus; pPHC = posterior parahippocampus cortex; preSMA = presupplementary motor area; SFG = superior frontal gyrus; SM = sensorimotor; SMA = supplementary motor area; SPL = superior parietal lobule

Table S3. Seed coordinates based on peak activation in clusters that were significantly associated with distraction and memory load effects in voxelwise analyses of all subjects.

| **Seed** | **X** | **Y** | **Z** |
| --- | --- | --- | --- |
| **Distraction: Encoding Phase** | | | |
| R SFG(BA 8) | 16 | 44 | 42 |
| R MFG(BA 6) | 27 | 2 | 57 |
| L MFG(BA 6) | -23 | 4 | 59 |
| R Precuneus | 26 | -64 | 48 |
| L Precuneus | -23 | -65 | 49 |
| L Lingual gyrus | -5 | -79 | -4 |
| R pPHC | 25 | -56 | -5 |
| L pPHC | -26 | -59 | -5 |
| R putamen | 21 | 10 | 3 |
| L Putamen | -21 | 12 | 3 |
| **Distraction: Retrieval Phase** | | | |
| L SMA | -8 | -19 | 55 |
| R SMA | 8 | -5 | 54 |
| R Precuneus | 29 | -66 | 48 |
| L Precuneus | -28 | -66 | 48 |
| L putamen | -25 | 9 | -2 |
| R putamen | 25 | 9 | -2 |
| **Memory Load: Encoding Phase** | | | |
| R MFG(BA 6) | 26 | -1 | 55 |
| L MFG(BA 6) | -27 | -1 | 55 |
| R DLPFC(BA 9) | 40 | 44 | 24 |
| R Precuneus | 29 | -66 | 48 |
| L Precuneus | -41 | -39 | 67 |
| R pPHC | 25 | -57 | -5 |
| L pPHC | -26 | -59 | -5 |
| R Caudate | 14 | 11 | 11 |
| R putamen | 24 | 9 | -2 |
| L Putamen | -25 | 9 | -2 |
| R mdThal | 12 | -18 | 7 |
| **Memory Load: Retrieval Phase** | | | |
| R MFG(BA 6) | 33 | 9 | 53 |
| L MFG(BA 6) | -35 | 9 | 53 |
| R DLPFC(BA 9) | 46 | 30 | 29 |
| L DLPFC(BA 9) | -44 | 20 | 27 |
| B preSMA | 0 | 20 | 51 |
| R Precuneus | 31 | -66 | 42 |
| L Precuneus | -29 | -68 | 39 |
| B PC | 0 | -44 | 27 |
| R putamen | 24 | 9 | -2 |
| L putamen | -25 | 9 | -2 |

X, Y, Z coordinates are base on the Montreal Neurological Institute (MNI) atlas.

BA = Brodmann area; L and R = left and right hemispheres; ant = anterior; post = posterior; BA = Brodmann area; DLPFC = dorsolateral prefrontal cortex; mdThal = mediodorsal thalamus; MFG = middle frontal gyrus; PC = posterior cingulate; pPHC = posterior parahippocampal cortex; SFG = superior frontal gyrus; preSMA = presupplementary motor area; SMA = supplementary motor area

**
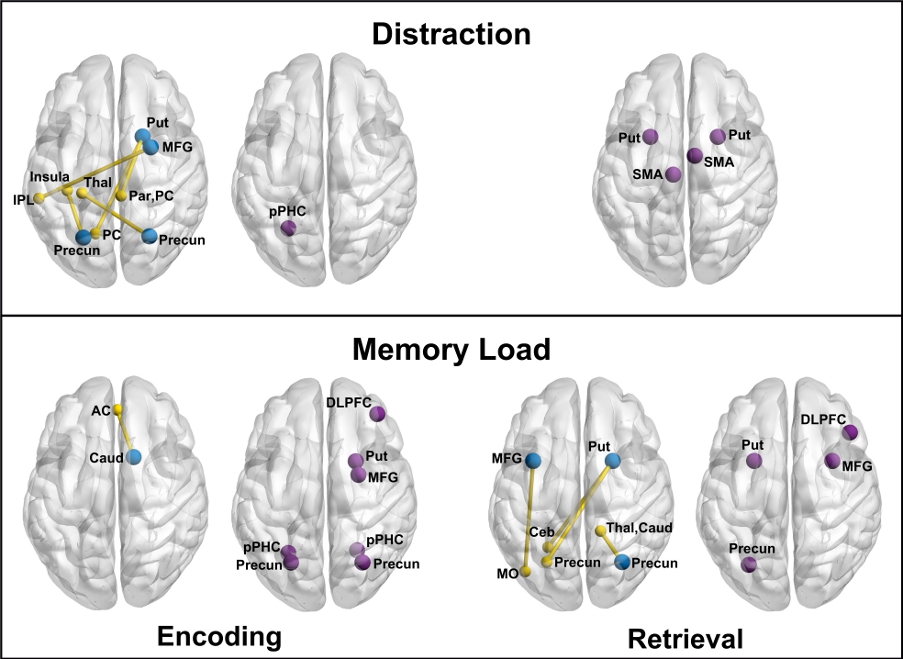
**

Figure S1. Regions of interest showing no group differences for distraction or memory load manipulations. The figure illustrates two different patterns of connectivity results that did not differ between the PD and control groups. Purple circles show seed ROI that did not exhibit distraction-related (top row) or memory load-related (bottom row) connectivity in either group (i.e., failed to reach the thresholded of a voxelwise-probability of p < 0.005 and minimum cluster size of 41 voxels). Blue circles display seed ROI that showed significant context-dependent with other brain regions (gold circles and lines), which did not differ between the PD and control groups. The top row shows that in both groups distraction dependent connectivity was greater during no distraction than distraction during the encoding phase. The bottom row shows that in both groups load-dependent connectivity was greater for 4 than 2 shape arrays during the encoding and retrieval phases. Tables 2 and 3 provide the coordinates for distraction and load-related connectivity features that did not differ between groups.

DLPFC = dorsal lateral prefrontal cortex; IPL = inferior parietal lobule; MFG = middle frontal gyrus (BA 6); MO = middle occipital (BA 19); Par = paracentral cortex; PC = posterior cingulate; Precun = precuneus; pPHC = posterior parahippocampal cortex; pPHC = posterior parahippocampal cortex; Put = putamen; SMA = supplementary motor area; Thal = thalamus
